# National, regional, and global prevalence of cigarette smoking among women/females in the general population: a systematic review and meta-analysis

**DOI:** 10.1186/s12199-020-00924-y

**Published:** 2021-01-08

**Authors:** Alireza Jafari, Abdolhalim Rajabi, Mahdi Gholian-Aval, Nooshin Peyman, Mehrsadat Mahdizadeh, Hadi Tehrani

**Affiliations:** 1 Department of Health Education and Health Promotion, Student Research Committee, Mashhad University of Medical Sciences, Mashhad, Iran; 2 Biostatistics and Epidemiology Department, Faculty of Health, Environmental Health Research Center, Golestan University of Medical Sciences, Gorgan, Iran; 3 Department of Health Education and Health Promotion, Social Determinants of Health Research Center, Mashhad University of Medical Sciences, Mashhad, Iran

**Keywords:** Cigarette smoking, Prevalence, Women, Tobacco, Nicotine

## Abstract

**Background:**

This systematic and meta-analysis review aimed to provide an updated estimate of the prevalence of ever and current cigarette smoking in women, in geographic areas worldwide, and demonstrate a trend of the prevalence of smoking over time by using a cumulative meta-analysis.

**Methods:**

Following PRISMA guidelines, we conducted a systematic review and meta-analysis of studies published on the prevalence of ever and current cigarette smoking in women. We searched PubMed, Web of Science (ISI), Scopus, and Ovid from January 2010 to April 2020. The reference lists of the studies included in this review were also screened. Data were reviewed and extracted independently by two authors. A random effects model was used to estimate the pooled prevalence of ever and current cigarette smoking in women. Sources of heterogeneity among the studies were determined using subgroup analysis and meta-regression.

**Results:**

The pooled prevalence of ever and current cigarette smoking in women was 28% and 17%, respectively. The pooled prevalence of ever cigarette smoking in adolescent girls/students of the school, adult women, pregnant women, and women with the disease was 23%, 27%, 32%, and 38%, respectively. The pooled prevalence of ever cigarette smoking in the continents of Oceania, Asia, Europe, America, and Africa was 36%, 14%, 38%, 31%, and 32%, respectively.

**Conclusions:**

The prevalence of cigarette smoking among women is very high, which is significant in all subgroups of adolescents, adults, and pregnant women. Therefore, it is necessary to design and implement appropriate educational programs for them, especially in schools, to reduce the side effects and prevalence of smoking among women.

**Supplementary Information:**

The online version contains supplementary material available at 10.1186/s12199-020-00924-y.

## Introduction

The prevalence of cigarette smoking among women has increased worldwide in recent years and is considered a public health concern [1]. Smoking is one of the most preventable causes of death from non-communicable diseases [2]. Smoking in women carries the risk of diseases such as cervical cancer, osteoporosis, cardiovascular disease, atherosclerosis, and type 2 diabetes, lung cancer, premature menopause, premature birth, abnormal fetal growth, low birth weight, miscarriage, and increases fetal death [3, 4]. Women who smoke before and during pregnancy increase the risk of preterm birth, abnormal fetal growth, low birth weight, miscarriage, and fetal death [5, 6].

World Health Organization (WHO) reported that one in ten deaths worldwide is caused by tobacco use, and that tobacco use worldwide causes 7 million deaths each year. If the world’s consumption patterns remain unchanged, by 2030, 8 million people will die from tobacco-related diseases every year [2, 7]. Cigarette smoking kills 480,000 people in the USA each year [8]. Every year, about 201,773 women around the world die from secondhand smoke [8].

Based on the results, 2000 adolescents under the age of 18 starts smoking every day for the first time in the world, and about 300 people start smoking daily [9]. The WHO reported that there are about 1.1 billion current cigarette smokers in the world [10]. The results of a meta-analysis study in China showed that the prevalence of smoking among women was 5.34% [11]. The results of a meta-analysis study in Iran showed that the prevalence of smoking among girls aged 12 to 17 years old was 6% [12]. A study conducted in Central and Eastern Europe showed that the prevalence of smoking among women was 64.7% [13].

One of the reasons for smoking in women has been the high number of tobacco companies promoting smoking by women in high and low-income countries over the past century. Advertising continues in low-income countries, where female smoking rates are still low. These companies have changed the cultural meaning of women’s smoking in society, removed cultural barriers to reduce the social pressure related to smoking, and increase the smoking rate of women [14].

The results have shown that smoking by family members, having smoking friends, and accompanying the family in smoking are other reasons for the increase in the prevalence of smoking in men and women [15]. Factors such as peer pressure, smoking attraction, testing, belonging to a consumer group, curiosity, and lack of appropriate options to reduce stress are influencing the onset and continuation of smoking by women [16]. Therefore, this study conducted a systematic review and meta-analysis to (1) provide an updated estimate of the prevalence of ever and current smoking in women worldwide, (2) explain the prevalence of smoking by geographical areas in the world, and (3) demonstrate a trend of the prevalence of smoking over time by using a collaborative meta-analysis.

## Methods

### Search strategy

**Fig. 1:**
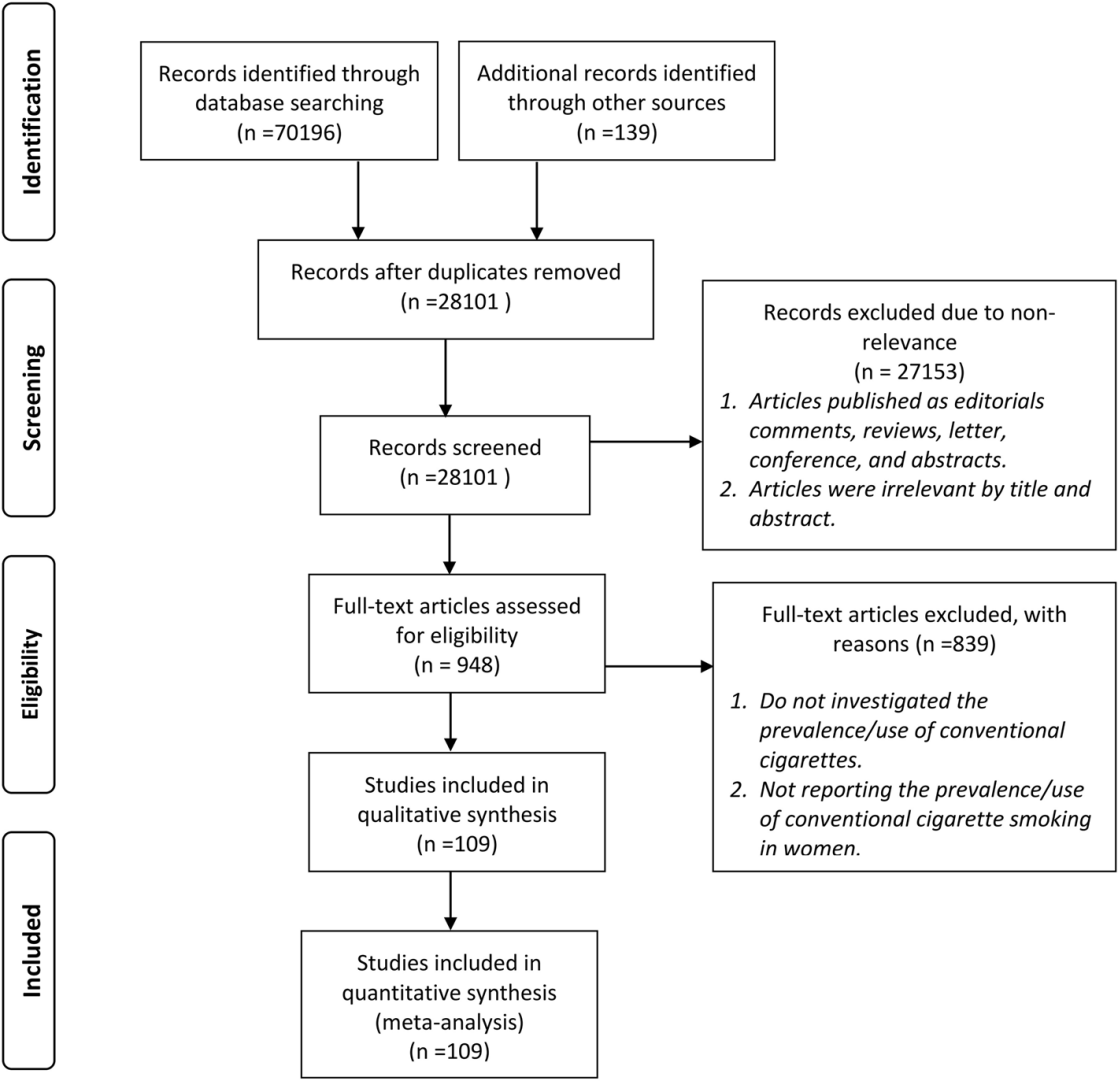
Flowchart of the systematic review process using PRISMA checklist

We searched the international electronic bibliographic databases including PubMed, Web of Science (ISI), Scopus, and Ovid from January 2010 to April 2020. Moreover, a manual search of the reference lists of the related articles was also performed, as well as the references of previous systematic reviews in the world were reviewed [17, 18]. This review was performed in accordance with the PRISMA (Preferred Reporting Items for Systematic Reviews and Meta-Analyses) statement issued in 2009 [19] and the GATHER guideline [20] (Fig. 1).

In this study, current smoking was defined as people who have smoked cigarettes at least once during the past 12 months, and ever smoking was defined as people who have smoked cigarettes during her/his lifetime. In our search strategy, we did apply limitations in the time of publication for January 2010 to April 2020 and English language. We reviewed the titles and abstracts to select potentially relevant papers. If there was doubt about the suitability of the paper in the abstract, the full-text was reviewed. We manually searched the references and relevant articles for inclusion. The search strategy is presented in Additional file 2. The protocol of this study was recorded in PROSPERO (record number: CRD42020183012).

### Study selection

All the records identified by the search were imported into the Endnote library, where duplicated publications were identified and excluded (Fig. 1). Similar to our previous systematic reviews [21], the remaining unique reports underwent two stages of screening, performed by AJ and AR. First, the titles and abstracts were screened, and those deemed to be relevant or potentially relevant were further screened, and then the full text of papers were searched and evaluated according to our inclusion and exclusion criteria. Eligible reports were included in this study, while other non-eligible reports were excluded for the reasons shown in Fig. 1.

### Inclusion and exclusion criteria

Studies were eligible for inclusion as follows: (1) studies published in the English language; (2) cross-sectional, cohort, and case-control studies reported data on smoking prevalence; (3) studies presented definitions of smoking; (4) if there were many studies based on the same sample, only the one that reported the most detailed data was included. Studies were excluded for the following reasons: (1) editorials, reviews, qualitative studies, commentary, conference abstracts, or presentations; (2) insufficient characterization of the methods; (3) lack of information necessary for the computation of prevalence from the articles or the authors (such as the number of samples or the prevalence rate of tobacco use). To avoid multiple publication bias in our meta-analysis, duplicate publications were excluded from the analysis.

### Quality assessment

The quality of the studies was evaluated by a validated quality assessment tool [22]. The tool included seven items that evaluated selection bias, measurement bias, and bias related to the analysis: (1) target population was defined clearly; (2) sampling is representative of potential respondents; (3) adequate response rate; (4) standardized data collection methods; (5) reliable survey instruments; (6) valid survey instruments; and (7) appropriate statistical methods. A total quality score was calculated based on the answers of “Yes” (scored 1) or “No” (scored 0) and varied between 0 and 7. All of the studies selected for this meta-analysis were assessed independently by two authors (AJ and AR). The discrepancies of the assessment results were resolved through discussion with a third author.

### Data extraction

The following data from all eligible studies were extracted: the first author, published year, year of study, study location, type of population, total sample size, the sample size in gender groups, smoking definition, and smoking prevalence. Data extraction was carried out by two authors (AJ and AR) independently. The discrepancies were resolved by discussion with another author.

### Data analysis

The extracted data were entered into the Excel software. Then, Stata 16.0 was used for analysis. Pooled prevalence and 95% confidence intervals were calculated using Der-Simonian and Laird method, taking into account conceptual heterogeneity, and I2, Tau2, and X2 were applied to assess heterogeneity between studies. The “metaprop” command was used to calculate the pooled prevalence of cigarette smoking and the prevalence in different subgroups, by available geographic regions, study settings, study population, and tools assessments of smoking. The pooled prevalence of tobacco smoking was presented in a forest plot, and the heterogeneity of studies conducted in each subgroup was estimated. The Q test was applied to assess heterogeneity between subgroups.

To assess differences in the accumulation of evidence for tobacco smoking prevalence, cumulative meta-analyses were conducted. The cumulative meta-analysis provides cumulative pooled estimates and 95% CIs. As studies are successively added, the overall prevalence and 95% CIs are recalculated providing evidence of the evolution of tobacco smoking prevalence over time. To assess the sequential contributions of studies and evaluate changes in tobacco smoking prevalence over time, studies were added alphabetically by years of implementation to a random-effects model. The sequential contributions of studies were evaluated in subgroups for tobacco smoking prevalence over time by cumulative meta-analysis.

## Results

In total, 70,335 articles were found by searching the databases. After deleting the duplicates articles (*n* = 42,234), articles were screened based on the title and abstract, and 27,153 articles were excluded because of not meeting the study criteria. In the next step, the full text of articles was assessed, and 839 articles that did not meet the inclusion criteria were removed from the study based on the reasons given in the flowchart. Finally, 109 articles entered the meta-analysis stage [23–131].

The prevalence of smoking was assessed in 18,290,793 women. The smallest and largest study in the present study was 131 and 14,912,100 women, respectively. The study includes 36 studies from the Americas, 34 studies from Asia, 27 studies from Europe, 8 studies from Africa, and 4 studies from the Oceania region. Other features are shown in Table S1.

**Fig. 2:**
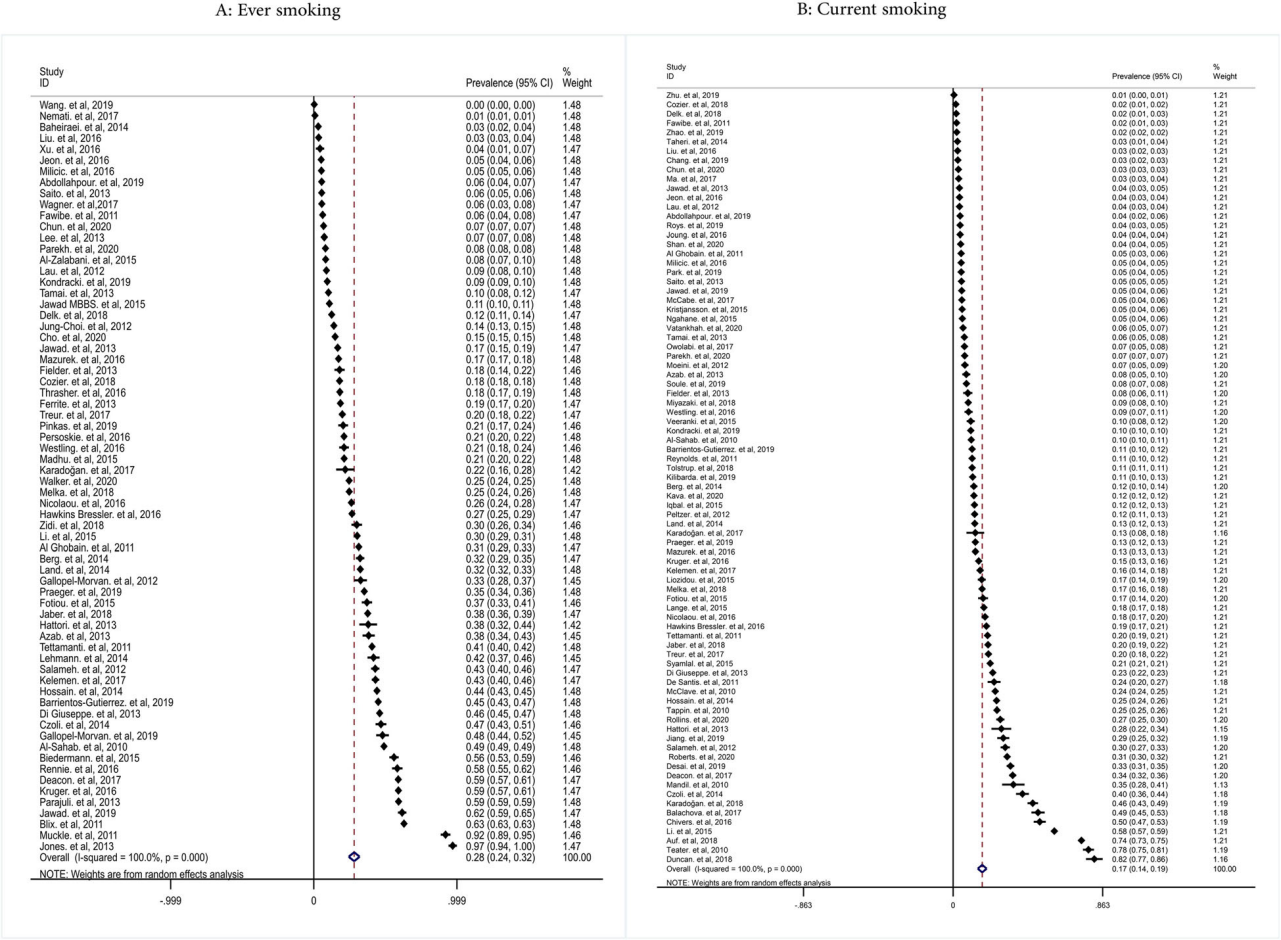
Pooled ever and current smoking prevalence in women

**Fig. 3:**
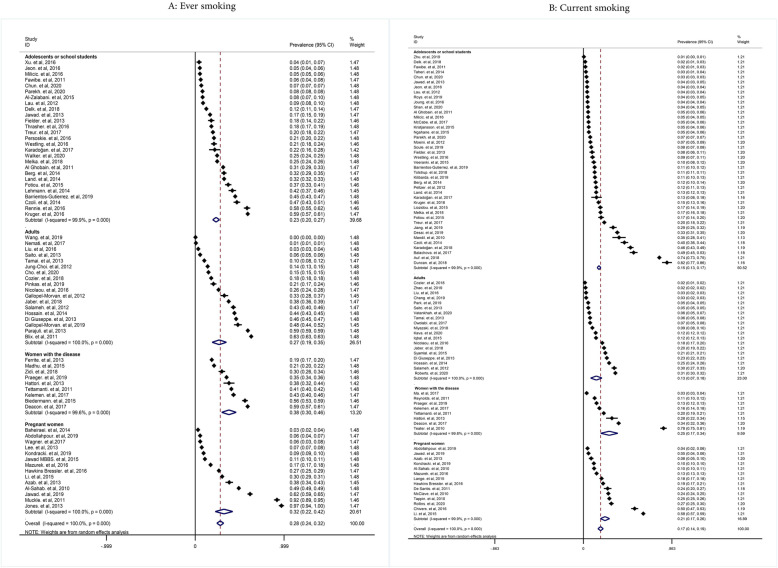
Pooled ever and current smoking prevalence in women by study population

The pooled prevalence of ever and current smoking in women was 28% (with confidence intervals (CIs) of 95%: 24-32% and 17% (95% CIs: 14-19%), respectively (Fig. 2)). The prevalence of ever cigarette smoking in adolescent girls/students of the school, adult women, pregnant women, and women with the disease was 23% (95% CIs: 20-27%), 27% (95%: 19-35%), 32% (95% CIs: 22-42%), and 38% (95% CIs: 30-46%), respectively. The pooled prevalence of current cigarette smoking in adolescent girls/students of the school, adult women, pregnant women, and women with the disease was 15% (95% CIs: 13-17%), 13% (0.95% CIs: 7-18%), 21% (95% CIs: 17-26%), and 25% (95% CIs: 17-34%), respectively (Fig. 3).

**Fig. 4:**
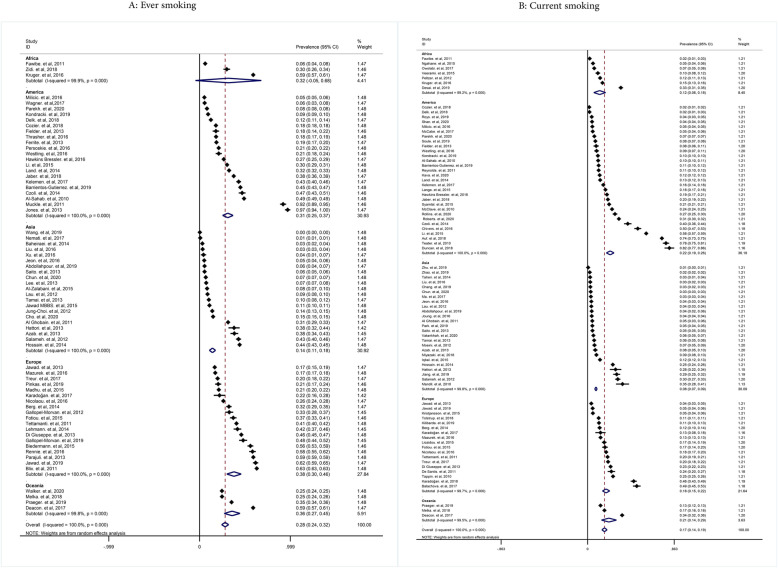
Pooled ever and current smoking prevalence in women by continent

The pooled prevalence of ever cigarette smoking according to the continents was as follow: Oceania 36% (95% CIs: 27-45%), Asia 14% (95% CIs: 11-18%), Europe 38% (95% CIs: 30-46%), America 31% (95% CIs: 25-37%), and Africa 32% (95% CIs: 5-68%). The pooled prevalence of current cigarette smoking according to the continents was for Oceania, Asia, Europe, America, and Africa 21% (95% CIs: 14-29%), 22% (95% CIs: 19-26%), 18% (95% CIs: 15-22%), 0.8% (95% CIs: 7-9%), and 12% (95% CIs: 6-18%), respectively (Fig. 4).

The pooled prevalence of ever cigarette smoking in cross-sectional studies, cohort studies, and case-control studies was 25% (95% CIs: 23-28%), 39% (95% CIs: 21-56%), and 34% (95% CIs: 21-47%), respectively. Also, the pooled prevalence of current cigarette smoking in cross-sectional studies, cohort studies, and case-control studies were 17% (95% CIs:14-20%), 14% (95% CIs: 10-19%), and 20% (95% CIs: 11-29%), respectively (Fig. S1).

According to the sampling method in the studies, the pooled prevalence of ever and current cigarette smoking in random sampling was 29% (95% CIs: 23-24%) and 14% (95% CIs: 11-18%), respectively (Fig. S2). According to the results of Fig. S3, by smoking assessment tools, the pooled prevalence of ever cigarette smoking in studies evaluated by the standard questionnaire and self-reporting was 35% (95% CIs: 24-46%), and 26% (95% CIs: 22-31%), respectively. In terms of smoking assessment tools, the prevalence of current smoking in studies evaluated by the standard questionnaire and self-reporting was 15% (95% CIs: 12-19%) and 17% (95% CIs: 14-20%), respectively (Fig. S3).

**Fig. 5:**
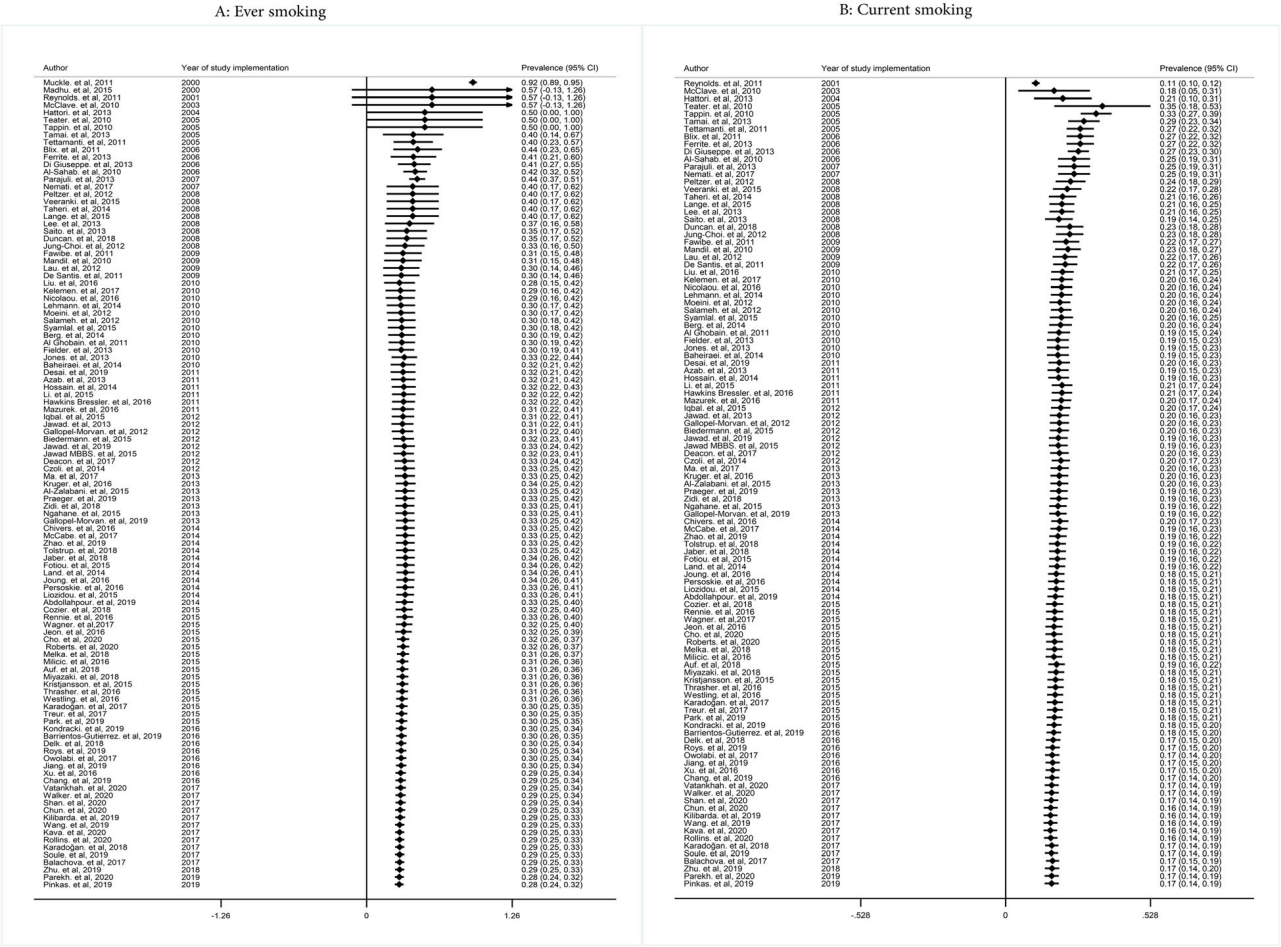
Cumulative meta-analysis of ever and current smoking prevalence among women

Cumulative meta-analysis test was conducted to examine the prevalence of ever smoking among women and increased the number of articles that have led to improved reading ability in recent years. The results showed that the prevalence of ever smoking has decreased from 2000 to 2010. So, in 2000, the prevalence of smoking was 57%, which in 2010 reached 30%. After 2010, the trend of the prevalence of smoking history has been almost constant. The trends were presented in different subgroups (Fig. 5). However, cumulative meta-analysis for current smoking showed that with the addition of studies conducted between 2001 and 2009, the trend of smoking prevalence in the current solution of cigarettes is increasing, then this trend is not clear until 2013, but after 2013 with the addition of new studies by 2019, the trend is declining (Fig. 5). The results of the prevalence trend of smoking by subgroups are shown in Figs. S4 and S8.

### Meta-regression analysis

The results of the univariate meta-regression analysis to investigating of sources of heterogeneity for the ever smoking showed that the years of the implementation study (*b* = −0.016, 95% CIs: −0.026, −0.005), continent Asia versus Africa (*b* = −0.17, 95% CIs: −0.29, −0.05) and study population women with disease versus adolescents or female students of the school (*b* = 0.14, 95% CIs: 0.01, 0.27) had a significant effect on ever smoking prevalence. Also, for current smoking showed that the years of the implementation study (*b* = −0.007, 95% CIs: −0.01, −0.003), continent America versus Africa (*b* = 0.10, 95% CIs: 0.03, 0.17), study population women with disease versus adolescents or school students (*b* = 0.09, 95% CIs: 0.0007, 0.19) had significant effect on current smoking prevalence.

## Discussion

This systematic review and meta-analysis study aimed to determine the global prevalence of cigarette smoking in women. The results of this study showed that the prevalence of ever and current smoking among women was 28% and 17%, respectively, which indicated that the prevalence of cigarette smoking among them is high. Based on the present study results, smoking has been declining from 2000 to 2019 and has decreased from 92% in 2000 to 28% in 2019. The WHO report showed that tobacco use by women worldwide has decreased from 10.5% in 2000 to 5.2% in 2020, which is consistent with the results of this study [132]. The prevalence of smoking in the subgroups of adolescents, adults, and pregnant women is mentioned below.

### The prevalence of cigarettes among girl students in schools

The present study showed that the prevalence of ever and current cigarette smoking among adolescent girls/female students of the school was 23% (95% CIs: 20-27%), and 15% (95% CIs: 13-17%), respectively. This means that the prevalence of ever and current smoking in this age group first showed an upward trend, then a downward trend at some point, and then almost a steady trend.

The WHO reported that the prevalence of smoking among girls under 15 in 2018 was 3.8% [132]. Based on the results of a systematic review study conducted by Xiong in China in 2020, the prevalence of smoking among female adolescents was 2.6% [18]. A study in 2019 showed that 64.7% of adolescent girls in European countries have ever smoked [13]. Various studies have reported a high prevalence of smoking among adolescents, which can be caused by smoking cessation rules for this age group in different countries, cultural and socio-economic differences, holding preventive programs in schools, educating parents in this regard, and not having easy access to smoking or there have been various restrictions on consumption in public places.

The results of various studies showed that factors such as smoking parents, low parental education, low-income family, low self-esteem, peer pressure, alcohol consumption, positive attitude toward smoking, having a smoking friend, actors smoking, parental divorce, or living with a parent were associated with smoking among adolescents [133–138].

An important issue for adolescents is that smoking is a major risk factor for future health, their tendency to use of cigarettes, alcohol, and drugs. The results of the study showed that adolescent smokers were seven times more likely to use smokeless tobacco, three times more likely to drink alcohol, and 10 to 30 times more likely to use drugs than nonsmokers [139]. Another study found that most smokers started cigarette smoking during their teenage years [140]. The results of a systematic review study showed that drug use was strongly associated with the onset of cigarette smoking [141]. On the other hand, smoking by adolescent girls causes various health problems for them and their fetus during pregnancy, and appropriate interventions must be taken to prevent smoking, especially among girls of school age, to reduce the prevalence of female smoking.

### The cigarette prevalence of adult women

Based on the results of this study, the prevalence of ever and current cigarette smoking in adult women was 27% (95%: 19-35), and 13% (0.95% CIs: 7-18), respectively. This means that the prevalence of ever and current smoking for this age group first showed an upward trend, then a downward trend at some point, and then almost a steady trend.

The WHO reported that the prevalence of cigarette smoking among girls over 15 years old in 2018 was 4.8% [132]. In a systematic review study conducted by Ding on Chinese women in 2014, it was reported that the prevalence of ever smoking was between 6 and 17%, and the prevalence of current smoking was between 1.4 and 5% [142]. A cohort study conducted in the USA showed that 31% of women smoked [88].

One of the reasons for the tendency of women to smoke is the advertising of the tobacco industry, which has recently targeted women and is trying to make more women more likely to smoke by advertising more [143]. Results of a systematic review study showed that factors such as having a smoking partner, job stress, and exposure to smoking are the factors related to smoking among women [142]. Other reasons for the onset of smoking by women include reduced stress, lower education levels, and lower prices for cigarettes [144, 145]. Increasing the prevalence of smoking in adult women increases various health problems such as the increased risk of stroke, cardiovascular disease, asthma, decreased lung function, breast cancer, cervical cancer, and cervical cancer [146–152]. Therefore, it is necessary to adopt the political and social laws to reduce access and smoking.

### The cigarette prevalence of pregnant women

Based on the results of this study, the prevalence of ever and current cigarette smoking among pregnant women was 32% (95% CIs: 22-42), and 21% (95% CIs:17-26%), respectively. This means that trend of ever smoking has been declining, but the trend of current smoking in pregnant women is almost constant, and the prevalence rate is still high. The results of the Lange study in 2018 showed that the global prevalence of smoking during pregnancy is 1.7%, which is lower than the prevalence of this study [17]. A study conducted by Kondracki in the USA showed that 9.5% of pregnant women smoked, of which 7% smoked during pregnancy [109]. The results of a 6-year study of women in Austria found that the prevalence of smoking was 18% [153].

One of the reasons for pregnant women’s tendency to smoke is insufficient knowledge about the effect of smoking on congenital anomalies [154]. Other reasons include physiological changes during pregnancy, long-term smoking before pregnancy, having a smoker partner, low education, low socioeconomic status, not attending in pregnancy classes, experiencing stressful events before pregnancy or during pregnancy, being depressed, having smoking friends, being in smoking environments, being exposed to secondhand smoke, unplanned pregnancy, starting smoking at an early age, high smoking intensity, being in lower social class during childhood, or early adulthood are effective factors in smoking during pregnancy [23, 155–158].

The prevalence of smoking in pregnant women is more important than other groups and due to the effects of smoking on the health of the pregnant women, and their fetus such as reduced fetal size, stillbirth, increased perinatal death, death infant, miscarriage, placental abruption, premature birth, premature lung aging, and chronic disease of obstructive pulmonary [159–162], it is necessary to pay more attention to this issue. Endangering the health of mother and fetus can affect the quality of life of family and society, and will have devastating consequences for the psychology, socioeconomic, and social aspects. The effects of mother smoking on the fetus can be devastating effects and endanger future generations of any society.

### The cigarette prevalence of the continents

The prevalence of ever smoking in the continents of Oceania, Asia, Europe, America, and Africa was 36% (95% CIs: 27-45%), 14% (95% CIs: 11-18%), 38% (95% CIs: 30-46%), 31% (95% CIs: 25-37%), and 32% (95% CIs: 5-68%), respectively.

The WHO report in 2018 showed that the prevalence of cigarette smoking among women in the continents of Africa, America, Eastern Mediterranean, Europe, Southeast Asia, and Western Pacific was 1.4%, 10.2%, 1.5%, 17.5%, 0.9, and 2.5%, respectively. The lowest prevalence is in the West Pacific and the Africa continent, while Europe has the highest prevalence [132]. Based on the WHO report, the cigarette smoking rate of women in European countries is higher than that of other countries in the world, which is consistent with the results of this study [163]. Based on the World Bank report, the prevalence of smoking among women in countries with high-income, upper middle-income, lower middle-income, and low-income was equal to 16.1%, 4.4%, 1.3%, and 2%, respectively, which indicating a high prevalence of consumption in high-income countries [132].

The results of a study have shown that the prevalence of smoking over the past 20 years has been gradually decreased in developed countries and steadily increasing in developing countries [164]. Results of a study showed that the price of cigarettes is declining in the middle- and low-income countries, and rising in the middle- and upper-income countries, and a 10% increase in cigarette prices has led to a 2% decrease in cigarette consumption [165]. The different prevalence rates in different continents and countries may be due to income levels, tax increases in certain countries, the implementation of various regional laws, and the implementation of education and prevention programs.

### The cigarette smoking prevalence by study design, sampling, and assessment tools

In the present study, most of the studies included were cross sectional. This study indicated that the prevalence of cigarette smoking in the cohort and cross-sectional studies were similar. The quality of cross-sectional and baseline cohort studies is usually more reliable and valid for estimating the prevalence [166], which was the same in the present study.

In estimating prevalence, the most appropriate sampling method is the random sampling method [167]. In the present study, the results are presented based on the subgroups and are valid and acceptable based on the random sampling method. Cigarette smoking usually is assessed by standard tools (such as questionnaires) or self-report. Usually, the data that is collected with standard tools is more reliable and valid [168]. In the present study, the subgroup analysis (standard tools and self-reporting tools) showed that the estimated prevalence in standard tools is somewhat different from self-reporting tools. The strengths of the present study were the determinant of the prevalence of ever and current cigarette smoking worldwide, in subgroups of adolescents, adults, pregnant women, and based on the continents.

## Limitations

Like other studies, this study had limitations. The first limitation was that in some studies data were collected using self-report tools which may cause biases. The second limitation was that in some studies the percentage or number of women smokers was not reported. The third limitation of this study was that the number of studies from the African continent was limited. The last limitation was that in some studies the prevalence of ever smoking or current smoking was not reported by women.

## Conclusion

Based on the results of the present study, the prevalence of cigarette smoking among women is very high, which is significant in all subgroups of adolescents, adults, and pregnant women. Given the role of women in the family, the growth and upbringing of children, it is essential to pay more attention to smoking in women. Therefore, it is necessary to design and implement appropriate educational programs for them, especially in schools, to reduce the side effects of smoking and reduce the prevalence of smoking among women.

## Supplementary Information

**Additional file 1: Table S1**. Population characteristics of the studies reported the prevalence of current and ever cigarette smoking among women.

**Additional file 2:.** Search Strategy.

**Additional file 3:.** PRISMA 2009 Checklist.

**Additional file 4: Fig S1**. Pooled ever and current smoking prevalence in women by study design.

**Additional file 5: Fig S2**. Pooled ever and current smoking prevalence in women by sampling method.

**Additional file 6: Fig S3**. Pooled ever and current smoking prevalence in women by tools assessment smoking.

**Additional file 7: Fig S4**. Cumulative meta-analysis of ever and current smoking prevalence among women by study population.

**Additional file 8: Fig S5**. Cumulative meta-analysis of ever and current smoking prevalence among women by continent.

**Additional file 9: Fig S6**. Cumulative meta-analysis of ever and current smoking prevalence among women by study design.

**Additional file 10: Fig S7**. Cumulative meta-analysis of ever and current smoking prevalence among women by sampling method.

**Additional file 11: Fig S8**. Cumulative meta-analysis of ever and current smoking prevalence among women tools assessment smoking.

## Data Availability

The data generated or analyzed during this study are available from the corresponding author on a reasonable request.

## References

[1] Hitchman SC, Fong GT (2011). Gender empowerment and female-to-male smoking prevalence ratios. Bull World Health Organ.

[2] Organization WH (2017). WHO report on the global tobacco epidemic, 2017: monitoring tobacco use and prevention policies.

[3] Islami F, Torre LA, Jemal A (2015). Global trends of lung cancer mortality and smoking prevalence. Transl Lung Cancer Res.

[4] Szkup M, Jurczak A, Karakiewicz B, Kotwas A, Kopeć J, Grochans E (2018). Influence of cigarette smoking on hormone and lipid metabolism in women in late reproductive stage. Clin Interv Aging.

[5] Acquavita SP, Talks A, Fiser K (2017). Facilitators and barriers to cigarette smoking while pregnant for women with substance use disorders. Nicotine Tob Res.

[6] Kurti AN, Bunn JY, Nighbor T, Cohen AH, Bolívar H, Tang KJ (2019). Leveraging technology to address the problem of cigarette smoking among women of reproductive age. Prev Med.

[7] Organization WH (2011). WHO report on the global tobacco epidemic, 2011: warning about the dangers of tobacco.

[8] Courtney R. The health consequences of smoking—50 years of progress: a report of the surgeon general, 2014 Us Department of Health and Human Services Atlanta, GA: Department of Health and Human Services, Centers for Disease Control and Prevention, National Center for Chronic Disease Prevention and Health Promotion, Office on Smoking and Health, 2014 1081 pp. Online (grey literature): http://www.surgeongeneral.gov/library/reports/50-years-of-progress. Drug Alcohol Rev 2015;34(6):694-695.

[9] Abuse S (2017). Mental Health Services Administration. Results from the 2016 National Survey on Drug Use and Health: detailed tables.

[10] Organization WH (2019). WHO report on the global tobacco epidemic 2019: offer help to quit tobacco use.

[11] Liu Y, Gao J, Shou J, Xia H, Shen Y, Zhu S (2016). The prevalence of cigarette smoking among rural-to-urban migrants in China: a systematic review and meta-analysis. Subst Use Misuse.

[12] Ehsani-Chimeh E, Sajadi HS, Behzadifar M, Aghaei M, Badrizadeh A, Behzadifar M (2020). Current and former smokers among adolescents aged 12–17 years in Iran: a systematic review and meta-analysis. BMC Public Health.

[13] Brożek GM, Jankowski M, Lawson JA, Shpakou A, Poznański M, Zielonka TM (2019). The prevalence of cigarette and e-cigarette smoking among students in Central and Eastern Europe—results of the YUPESS study. Int J Environ Res Public Health.

[14] Greaves L (2015). The meanings of smoking to women and their implications for cessation. Int J Environ Res Public Health.

[15] Johnston V, Westphal DW, Earnshaw C, Thomas DP (2012). Starting to smoke: a qualitative study of the experiences of Australian indigenous youth. BMC Public Health.

[16] Salvi D, Nagarkar A (2018). A qualitative study exploring women´s journeys to becoming smokers in the social context of urban India. Tob Induc Dis.

[17] Lange S, Probst C, Rehm J, Popova S (2018). National, regional, and global prevalence of smoking during pregnancy in the general population: a systematic review and meta-analysis. Lancet Glob Health.

[18] Xiong PS, Xiong MJ, Liu ZX, Liu Y (2020). Prevalence of smoking among adolescents in China: an updated systematic review and meta-analysis. Public Health.

[19] Moher D, Liberati A, Tetzlaff J, Altman DG (2009). Preferred reporting items for systematic reviews and meta-analyses: the PRISMA statement. PLoS Med.

[20] Stevens GA, Alkema L, Black RE, Boerma JT, Collins GS, Ezzati M (2016). Correction: guidelines for accurate and transparent health estimates reporting: the GATHER statement. PLoS Med.

[21] Rajabi A, Dehghani M, Shojaei A, Farjam M, Motevalian SA (2019). Association between tobacco smoking and opioid use: A meta-analysis. Addict Behav.

[22] Boyle MH (1998). Guidelines for evaluating prevalence studies. Evid Based Mental Health.

[23] Al-Sahab B, Saqib M, Hauser G, Tamim H (2010). Prevalence of smoking during pregnancy and associated risk factors among Canadian women: a national survey. BMC Pregnancy Childbirth.

[24] McClave AK, Hogue CJ, Huber LRB, Ehrlich AC (2010). Cigarette smoking women of reproductive age who use oral contraceptives: results from the 2002 and 2004 behavioral risk factor surveillance systems. Womens Health Issues.

[25] Tappin DM, MacAskill S, Bauld L, Eadie D, Shipton D, Galbraith L (2010). Smoking prevalence and smoking cessation services for pregnant women in Scotland. Subst Abuse Treat Prevent Policy.

[26] Mandil A, BinSaeed A, Ahmad S, Al-Dabbagh R, Alsaadi M, Khan M (2010). Smoking among university students: a gender analysis. J Infect Public Health.

[27] Al Ghobain MO, Al Moamary MS, Al Shehri SN, AL-Hajjaj MS (2011). Prevalence and characteristics of cigarette smoking among 16 to 18 years old boys and girls in Saudi Arabia. Ann Thoracic Med.

[28] Fawibe A, Shittu A (2011). Prevalence and characteristics of cigarette smokers among undergraduates of the University of Ilorin, Nigeria. Niger J Clin Pract.

[29] Reynolds K, Liese AD, Anderson AM, Dabelea D, Standiford D, Daniels SR (2011). Prevalence of tobacco use and association between cardiometabolic risk factors and cigarette smoking in youth with type 1 or type 2 diabetes mellitus. J Pediatr.

[30] Blix HS, Hjellvik V, Litleskare I, Rønning M, Tverdal A (2011). Cigarette smoking and risk of subsequent use of antibacterials: a follow-up of 365 117 men and women. J Antimicrob Chemother.

[31] De Santis M, De Luca C, Mappa I, Quattrocchi T, Angelo L, Cesari E (2011). Smoke, alcohol consumption and illicit drug use in an Italian population of pregnant women. Eur J Obstet Gynecol Reprod Biol.

[32] Muckle G, Laflamme D, Gagnon J, Boucher O, Jacobson JL, Jacobson SW (2011). Alcohol, smoking, and drug use among Inuit women of childbearing age during pregnancy and the risk to children. Alcohol Clin Exp Res.

[33] Tettamanti G, Nyman-Iliadou A, Pedersen NL, Bellocco R, Milsom I, Altman D (2011). Influence of smoking, coffee, and tea consumption on bladder pain syndrome in female twins. Urology..

[34] Lau M, Chen X, Ren Y (2012). Increased risk of cigarette smoking among immigrant children and girls in Hong Kong: an emerging public health issue. J Community Health.

[35] Salameh P, Khayat G, Waked M (2012). Lower prevalence of cigarette and waterpipe smoking, but a higher risk of waterpipe dependence in Lebanese adult women than in men. Women Health.

[36] Peltzer K (2012). Prevalence, Correlates and Perceptions Towards Cigarette Smoking Among Male and Female in School Adolescents (Aged 11–18 Years) in South Africa: Results from the 2008 GYTS Study. J Psychol Afr.

[37] Gallopel-Morvan K, Moodie C, Hammond D, Eker F, Beguinot E, Martinet Y (2012). Consumer perceptions of cigarette pack design in France: a comparison of regular, limited edition and plain packaging. Tob Control.

[38] Moeini B, Poorolajal J, Gharghani ZG (2012). Prevalence of cigarette smoking and associated risk factors among adolescents in Hamadan City, west of Iran in 2010. J Res Health Sci.

[39] Jung-Choi K-H, Khang Y-H, Cho H-J (2012). Hidden female smokers in Asia: a comparison of self-reported with cotinine-verified smoking prevalence rates in representative national data from an Asian population. Tob Control.

[40] Fielder RL, Carey KB, Carey MP (2013). Hookah, cigarette, and marijuana use: a prospective study of smoking behaviors among first-year college women. Addict Behav.

[41] Jones HE, Heil SH, Tuten M, Chisolm MS, Foster JM, O’Grady KE (2013). Cigarette smoking in opioid-dependent pregnant women: neonatal and maternal outcomes. Drug Alcohol Depend.

[42] Hattori T, Konno S, Shijubo N, Ohmichi M, Nishimura M (2013). Increased prevalence of cigarette smoking in J apanese patients with sarcoidosis. Respirology..

[43] Jawad M, Wilson A, Lee JT, Jawad S, Hamilton FL, Millett C (2013). Prevalence and predictors of water pipe and cigarette smoking among secondary school students in London. Nicotine Tob Res.

[44] Tamai Y, Tsuji M, Wada K, Nakamura K, Hayashi M, Takeda N (2014). Association of cigarette smoking with skin colour in Japanese women. Tob Control.

[45] Di Giuseppe D, Orsini N, Alfredsson L, Askling J, Wolk A (2013). Cigarette smoking and smoking cessation in relation to risk of rheumatoid arthritis in women. Arthritis Res Ther.

[46] Ferrite S, Santana VS, Marshall SW (2013). Interaction between noise and cigarette smoking for the outcome of hearing loss among women: A population-based study. Am J Ind Med.

[47] Parajuli R, Bjerkaas E, Tverdal A, Selmer R, Le Marchand L, Weiderpass E (2013). The increased risk of colon cancer due to cigarette smoking may be greater in women than men. Cancer Epidemiol Prevent Biomarkers.

[48] Saito N, Sairenchi T, Irie F, Iso H, Iimura K, Watanabe H (2013). Duration of cigarette smoking is a risk factor for oropharyngeal cancer mortality among Japanese men and women: the Ibaraki Prefectural Health Study (IPHS). Ann Eepidemiol.

[49] Lee J-Y, Ko Y-J, Park S (2013). Factors associated with current smoking and heavy alcohol consumption among women of reproductive age: the Fourth Korean National Health and Nutrition Examination Survey 2007–2009. Public Health.

[50] Azab M, Khabour OF, Alzoubi KH, Anabtawi MM, Quttina M, Khader Y (2012). Exposure of pregnant women to waterpipe and cigarette smoke. Nicotine Tob Res.

[51] Baheiraei A, Mirghafourvand M, Mohammad-Alizadeh Charandabi S, NEDjAt S, Mohammadi E (2014). A population-based survey on prevalence of cigarette smoking and its socio-demographic risk factors among women of reproductive age in Tehran-Iran. Epidemiol Biostat Public Health.

[52] Land SR, Liu Q, Wickerham DL, Costantino JP, Ganz PA (2014). Cigarette smoking, physical activity, and alcohol consumption as predictors of cancer incidence among women at high risk of breast cancer in the NSABP P-1 trial. Cancer Epidemiol Prevent Biomarkers.

[53] Czoli CD, Hammond D, White CM (2014). Electronic cigarettes in Canada: prevalence of use and perceptions among youth and young adults. Can J Public Health.

[54] Lehmann F, von Lindeman K, Klewer J, Kugler J (2014). BMI, physical inactivity, cigarette and alcohol consumption in female nursing students: a 5-year comparison. BMC Med Educ.

[55] Berg CJ, Aslanikashvili A, Djibuti M (2014). A cross-sectional study examining youth smoking rates and correlates in Tbilisi, Georgia. Biomed Res Int.

[56] Hossain MS, Kypri K, Rahman B, Arslan I, Akter S, Milton AH (2014). Prevalence and correlates of smokeless tobacco consumption among married women in rural Bangladesh. PLoS One.

[57] Jawad M, Abdulrahim S, Daouk A (2015). The social patterning of tobacco use among women in Jordan: the protective effect of education on cigarette smoking and the deleterious effect of wealth on cigarette and waterpipe smoking. Nicotine Tob Res.

[58] Madhu C, Enki D, Drake MJ, Hashim H (2015). The functional effects of cigarette smoking in women on the lower urinary tract. Urol Int.

[59] Al-Zalabani A, Kasim K (2015). Prevalence and predictors of adolescents’ cigarette smoking in Madinah, Saudi Arabia: a school-based cross-sectional study. BMC Public Health.

[60] Fotiou A, Kanavou E, Stavrou M, Richardson C, Kokkevi A (2015). Prevalence and correlates of electronic cigarette use among adolescents in Greece: a preliminary cross-sectional analysis of nationwide survey data. Addict Behav.

[61] Ngahane BHM, Ekobo HA, Kuaban C (2015). Prevalence and determinants of cigarette smoking among college students: a cross-sectional study in Douala, Cameroon. Arch Public Health.

[62] Liozidou A, Dimou N, Lioupa A, Behrakis P (2015). Prevalence and predictors of cigarette smoking among Greek urban adolescents: A cross-sectional study. Tob Prev Cessat.

[63] Syamlal G, Mazurek JM, Storey E, Dube SR (2015). Cigarette smoking prevalence among adults working in the health care and social assistance sector, 2008 to 2012. J Occup Environ Med/Am College Occup Environ Med.

[64] Biedermann L, Fournier N, Misselwitz B, Frei P, Zeitz J, Manser CN (2015). High rates of smoking especially in female Crohn’s disease patients and low use of supportive measures to achieve smoking cessation—data from the Swiss IBD cohort study. J Crohn's Colitis.

[65] Lange S, Probst C, Quere M, Rehm J, Popova S (2015). Alcohol use, smoking and their co-occurrence during pregnancy among Canadian women, 2003 to 2011/12. Addict Behav.

[66] Li X (2015). Sociodemographic and psychological characteristics of very light smoking among women in emerging adulthood, national survey of drug use and health, 2011. Prev Chronic Dis.

[67] Taheri E, Ghorbani A, Salehi M, Sadeghnia HR (2015). Cigarette smoking behavior and the related factors among the students of mashhad university of medical sciences in iran. Iran Red Crescent Med J.

[68] Veeranki SP, Mamudu HM, John RM, Ouma AE (2015). Prevalence and correlates of tobacco use among school-going adolescents in Madagascar. J Epidemiol Global Health.

[69] Iqbal N, Irfan M, Ashraf N, Awan S, Khan JA (2015). Prevalence of tobacco use among women: a cross sectional survey from a squatter settlement of Karachi, Pakistan. BMC Res Notes.

[70] Kristjansson AL, Mann MJ, Sigfusdottir ID (2015). Licit and illicit substance use by adolescent e-cigarette users compared with conventional cigarette smokers, dual users, and nonusers. J Adolesc Health.

[71] Chivers LL, Hand DJ, Priest JS, Higgins ST (2016). E-cigarette use among women of reproductive age: Impulsivity, cigarette smoking status, and other risk factors. Prev Med.

[72] Mazurek JM, England LJ (2016). Cigarette smoking among working women of reproductive age—United States, 2009–2013. Nicotine Tob Res.

[73] Thrasher JF, Abad-Vivero EN, Barrientos-Gutíerrez I, Pérez-Hernández R, Reynales-Shigematsu LM, Mejía R (2016). Prevalence and correlates of e-cigarette perceptions and trial among early adolescents in Mexico. J Adolesc Health.

[74] Westling E, Rusby JC, Crowley R, Light JM. Electronic cigarette use by youth: prevalence, correlates, and use trajectories from middle to high school. J Adolesc Health 2017;60(6):660-6. 10.1016/j.jadohealth.2016.12.019 PMC5441946 28242187

[75] Rennie LJ, Bazillier-Bruneau C, Rouëssé J (2016). Harm reduction or harm introduction? Prevalence and correlates of e-cigarette use among French adolescents. J Adolesc Health.

[76] Bressler LH, Bernardi LA, De Chavez PJD, Baird DD, Carnethon MR, Marsh EE (2016). Alcohol, cigarette smoking, and ovarian reserve in reproductive-age African-American women. Am J Obstet Gynecol.

[77] Jeon C, Jung KJ, Kimm H, Lee S, Barrington-Trimis JL, McConnell R (2016). E-cigarettes, conventional cigarettes, and dual use in Korean adolescents and university students: Prevalence and risk factors. Drug Alcohol Depend.

[78] Xu Y, Chen X (2016). Protection motivation theory and cigarette smoking among vocational high school students in China: a cusp catastrophe modeling analysis. Global Health Res Policy.

[79] Joung MJ, Han MA, Park J, Ryu SY (2016). Association between family and friend smoking status and adolescent smoking behavior and e-cigarette use in Korea. Int J Environ Res Public Health.

[80] Persoskie A, Donaldson EA, King BA (2016). Peer Reviewed: Ever-Use and Curiosity About Cigarettes, Cigars, Smokeless Tobacco, and Electronic Cigarettes Among US Middle and High School Students, 2012–2014. Prev Chronic Dis.

[81] Kruger L, van Walbeek C, Vellios N (2016). Waterpipe and cigarette smoking among university students in the Western Cape, South Africa. Am J Health Behav.

[82] Nicolaou SA, Heraclides A, Markides K, Charalambous A (2016). Prevalence and social determinants of smoking in the adult Greek Cypriot population. Hippokratia..

[83] Liu S, Zhang M, Yang L, Li Y, Wang L, Huang Z (2017). Prevalence and patterns of tobacco smoking among Chinese adult men and women: findings of the 2010 national smoking survey. J Epidemiol Community Health.

[84] Ma Y, Wen L, Cui W, Yuan W, Yang Z, Jiang K (2017). Prevalence of cigarette smoking and nicotine dependence in men and women residing in two provinces in China. Front Psychiatry.

[85] Wagner NJ, Camerota M, Propper C (2017). Prevalence and perceptions of electronic cigarette use during pregnancy. Matern Child Health J.

[86] McCabe SE, West BT, Veliz P, Boyd CJ (2017). E-cigarette use, cigarette smoking, dual use, and problem behaviors among US adolescents: results from a national survey. J Adolesc Health.

[87] Milicic S, Leatherdale ST (2017). The associations between e-cigarettes and binge drinking, marijuana use, and energy drinks mixed with alcohol. J Adolesc Health.

[88] Kelemen LE, Abbott S, Qin B, Peres LC, Moorman PG, Wallace K (2017). Cigarette smoking and the association with serous ovarian cancer in African American women: African American Cancer Epidemiology Study (AACES). Cancer Causes Control.

[89] Nemati S, Rafei A, Freedman ND, Fotouhi A, Asgary F, Zendehdel K (2017). Cigarette and water-pipe use in Iran: geographical distribution and time trends among the adult population; a pooled analysis of national STEPS surveys, 2006–2009. Arch Iran Med.

[90] Karadoğan D, Önal Ö, Say Şahin D, Yazıcı S, Kanbay Y (2017). Evaluation of school teachers’ sociodemographic characteristics and quality of life according to their cigarette smoking status: a cross-sectional study from eastern Black Sea region of Turkey. Eur Respir J.

[91] Balachova T, Zander R, Bonner B, Isurina G, Kyler K, Tsvetkova L (2019). Smoking and alcohol use among women in Russia: Dual risk for prenatal exposure. J Ethn Subst Abus.

[92] Deacon RM, Mooney-Somers J (2017). Smoking prevalence among lesbian, bisexual and queer women in Sydney remains high: Analysis of trends and correlates. Drug Alcohol Rev.

[93] Owolabi E, Goon D, Adeniyi O, Seekoe E, Adedokun A (2017). Prevalence and factors associated with tobacco use among adults attending selected healthcare facilities in Buffalo City Metropolitan Municipality, South Africa. S Afr Fam Pract.

[94] Auf R, Trepka MJ, Selim M, Taleb ZB, De La Rosa M, Bastida E (2019). E-cigarette use is associated with other tobacco use among US adolescents. Int J Public Health.

[95] Jaber RM, Mirbolouk M, DeFilippis AP, Maziak W, Keith R, Payne T (2018). Electronic cigarette use prevalence, associated factors, and pattern by cigarette smoking status in the United States from NHANES (National Health and Nutrition Examination Survey) 2013–2014. J Am Heart Assoc.

[96] Cozier YC, Barbhaiya M, Castro-Webb N, Conte C, Tedeschi SK, Leatherwood C (2019). Relationship of cigarette smoking and alcohol consumption to incidence of systemic lupus erythematosus in a prospective cohort study of black women. Arthritis Care Res.

[97] Melka AS, Chojenta CL, Holliday EG, Loxton DJ (2019). Predictors of E-cigarette use among young Australian women. Am J Prev Med.

[98] Zidi S, Sahli M, Mezlini A, Yacoubli-Loueslati B (2020). Association of Combined Tobacco Smoking, Hormonal Contraceptive use and Status Matrimonial with Cervical Cancer Evolution in Tunisian Women. POR..

[99] Duncan K, Erickson AC, Egeland GM, Weiler H, Arbour LT (2018). Red blood cell folate levels in Canadian Inuit women of childbearing years: influence of food security, body mass index, smoking, education, and vitamin use. Can J Public Health.

[100] Miyazaki Y, Tabuchi T (2018). Educational gradients in the use of electronic cigarettes and heat-not-burn tobacco products in Japan. PLoS One.

[101] Treur JL, Rozema AD, Mathijssen JJ, van Oers H, Vink JM (2018). E-cigarette and waterpipe use in two adolescent cohorts: cross-sectional and longitudinal associations with conventional cigarette smoking. Eur J Eepidemiol.

[102] Tolstrup JS, Pisinger VSC, Egan KK, Christensen AI (2018). Trends in smoking and smokeless tobacco use among Danish Adolescents, 1997-2014. Tob Prev Cessat.

[103] Delk J, Creamer MR, Perry CL, Harrell MB (2018). Weight status and cigarette and electronic cigarette use in adolescents. Am J Prev Med.

[104] Karadoğan D, Önal Ö, Kanbay Y (2018). Prevalence and determinants of smoking status among university students: Artvin Çoruh University sample. PLoS One.

[105] Abdollahpour I, Mansournia MA, Salimi Y, Nedjat S (2019). Lifetime prevalence and correlates of smoking behavior in Iranian adults’ population; a cross-sectional study. BMC Public Health.

[106] Wang R, Jiang Y, Yao C, Zhu M, Zhao Q, Huang L (2019). Prevalence of tobacco related chronic diseases and its role in smoking cessation among smokers in a rural area of Shanghai, China: a cross sectional study. BMC Public Health.

[107] Pinkas J, Kaleta D, Zgliczyński WS, Lusawa A, Wrześniewska-Wal I, Wierzba W (2019). The prevalence of tobacco and E-cigarette use in Poland: A 2019 Nationwide Cross-Sectional Survey. Int J Environ Res Public Health.

[108] Kondracki AJ (2019). Prevalence and patterns of cigarette smoking before and during early and late pregnancy according to maternal characteristics: the first national data based on the 2003 birth certificate revision, United States, 2016. Reprod Health.

[109] Zhu J, Shi F, Xu G, Li N, Li J, He Y (2019). Conventional cigarette and E-cigarette smoking among school personnel in shanghai, China: Prevalence and determinants. Int J Environ Res Public Health.

[110] Desai R, Mercken LA, Ruiter RA, Schepers J, Reddy PS (2019). Cigarette smoking and reasons for leaving school among school dropouts in South Africa. BMC Public Health.

[111] Gallopel-Morvan K, Moodie C, Guignard R, Eker F, Béguinot E (2019). Consumer perceptions of cigarette design in France: a comparison of regular, slim, pink and plain cigarettes. Nicotine Tob Res.

[112] Jawad A, Patel D, Brima N, Stephenson J (2019). Alcohol, smoking, folic acid and multivitamin use among women attending maternity care in London: a cross-sectional study. Sex Reprod Healthc.

[113] Praeger R, Roxburgh A, Passey M, Mooney-Somers J (2019). The prevalence and factors associated with smoking among lesbian and bisexual women: Analysis of the Australian National Drug Strategy Household Survey. Int J Drug Policy.

[114] Barrientos-Gutierrez I, Lozano P, Arillo-Santillan E, Morello P, Mejia R, Thrasher JF (2019). “Technophilia”: A new risk factor for electronic cigarette use among early adolescents?. Addict Behav.

[115] Park MB, Choi JK (2019). Differences between the effects of conventional cigarettes, e-cigarettes and dual product use on urine cotinine levels. Tob Induc Dis.

[116] Kilibarda B, Vukovic D, Krstev S (2019). Prevalence and correlates of concurrent use of cigarettes, electronic cigarettes, and waterpipes among Serbian youth. Tob Induc Dis.

[117] Chang Y, Kang H-Y, Lim D, Cho H-J, Khang Y-H (2019). Long-term trends in smoking prevalence and its socioeconomic inequalities in Korea, 1992–2016. Int J Equity Health.

[118] Soule EK, Rossheim ME, Cavazos TC, Bode K, Desrosiers AC. Cigarette, waterpipe, and electronic cigarette use among college fraternity and sorority members and athletes in the United States. J Am Coll Heal. 2019:1–7. 10.1080/07448481.2019.1680555 PMC7205588 31702957

[119] Zhao L, Mbulo L, Palipudi K, Wang J, King B (2019). Awareness and use of e-cigarettes among urban residents in China. Tob Induc Dis.

[120] Jiang N, Cleland CM, Wang MP, Kwong A, Lai V, Lam TH (2019). Perceptions and use of e-cigarettes among young adults in Hong Kong. BMC Public Health.

[121] Rollins LG, Sokol NA, McCallum M, England L, Matteson K, Werner E (2020). Electronic Cigarette Use During Preconception and/or Pregnancy: Prevalence, Characteristics, and Concurrent Mental Health Conditions. J Women's Health.

[122] Cho B-Y, Lin H-C, Seo D-C (2020). Effectiveness of Indiana’s Statewide Smoke-Free Indoor Air Law in Reducing Prevalence of Adult Cigarette Smoking. J Prim Prev.

[123] Vatankhah S, Naghdi S, Ghiasvand H, Armoon B, Ahounabr E (2020). Current cigarette smoking among Iranian elders; what are the prevalence, inequality and socioeconomic determinants? An analysis on Iranian Rural and Urban Income-Expenditure Survey 2017. J Addict Dis.

[124] Walker N, Parag V, Wong SF, Youdan B, Broughton B, Bullen C (2020). Use of e-cigarettes and smoked tobacco in youth aged 14-15 years in New Zealand: findings from repeated cross-sectional studies (2014-19). Lancet Public Health.

[125] Parekh T, Pemmasani S, Desai R (2020). Risk of stroke with e-cigarette and combustible cigarette use in young adults. Am J Prev Med.

[126] Shan L, Manzione LC, Azagba S (2020). Psychological well-being and dual-use of cigarettes and e-cigarettes among high school students in Canada. J Affect Disord.

[127] Chun J, Yu M, Kim J, Kim A (2020). E-Cigarette, Cigarette, and Dual Use in Korean Adolescents: A Test of Problem Behavior Theory. J Psychoactive Drugs.

[128] Roys MR, Peltier MR, Stewart SA, Waters AF, Waldo KM, Copeland AL (2020). The association between problematic alcohol use, risk perceptions, and e-cigarette use. Am J Drug Alcohol Abuse.

[129] Roberts W, Verplaetse T, Peltier MKR, Moore KE, Gueorguieva R, McKee SA (2020). Prospective association of e-cigarette and cigarette use with alcohol use in two waves of the Population Assessment of Tobacco and Health. Addiction..

[130] Kava CM, Hannon PA, Harris JR (2020). Use of cigarettes and E-cigarettes and dual use among adult employees in the US workplace. Prev Chronic Dis.

[131] Teater B, Hammond GC (2010). Exploring smoking prevalence, quit attempts, and readiness to quit cigarette use among women in substance abuse treatment. Soc Work Health Care.

[132] Organization WH (2019). WHO global report on trends in prevalence of tobacco smoking 2000–2025.

[133] Oyewole BK, Animasahun VJ, Chapman HJ (2018). Tobacco use in Nigerian youth: A systematic review. PLoS One.

[134] Wang M, Zhong J-M, Fang L, Wang H (2016). Prevalence and associated factors of smoking in middle and high school students: a school-based cross-sectional study in Zhejiang Province, China. BMJ Open.

[135] Joffer J, Burell G, Bergström E, Stenlund H, Sjörs L, Jerdén L (2014). Predictors of smoking among Swedish adolescents. BMC Public Health.

[136] Leshargie CT, Alebel A, Kibret GD, Birhanu MY, Mulugeta H, Malloy P (2019). The impact of peer pressure on cigarette smoking among high school and university students in Ethiopia: A systemic review and meta-analysis. PLoS One.

[137] Dereje N, Abazinab S, Girma A (2014). Prevalence and predictors of cigarette smoking among adolescents of Ethiopia: school based cross sectional survey. J Child Adolesc Behav.

[138] Oh DL, Heck JE, Dresler C, Allwright S, Haglund M, Del Mazo SS (2010). Determinants of smoking initiation among women in five European countries: a cross-sectional survey. BMC Public Health.

[139] Torabi MR, Bailey WJ, Majd-Jabbari M (1993). Cigarette smoking as a predictor of alcohol and other drug use by children and adolescents: evidence of the "gateway drug effect". J Sch Health.

[140] Seo DC, Huang Y (2012). Systematic review of social network analysis in adolescent cigarette smoking behavior. J Sch Health.

[141] Freedman KS, Nelson NM, Feldman LL (2012). Smoking initiation among young adults in the United States and Canada, 1998-2010: a systematic review. Prev Chronic Dis.

[142] Ding D, Gebel K, Oldenburg BF, Wan X, Zhong X, Novotny TE (2014). An early-stage epidemic: a systematic review of correlates of smoking among Chinese women. Int J Behav Med.

[143] Barbeau EM, Leavy-Sperounis A, Balbach E (2004). Smoking, social class, and gender: what can public health learn from the tobacco industry about disparities in smoking?. Tob Control.

[144] Aniwada EC, Uleanya ND, Ossai EN, Nwobi EA, Anibueze M (2018). Tobacco use: prevalence, pattern, and predictors, among those aged 15-49 years in Nigeria, a secondary data analysis. Tob Induc Dis.

[145] Torres OV, O'Dell LE (2016). Stress is a principal factor that promotes tobacco use in females. Prog Neuro-Psychopharmacol Biol Psychiatry.

[146] Aryanpur M, Yousefifard M, Oraii A, Heydari G, Kazempour-Dizaji M, Sharifi H (2019). Effect of passive exposure to cigarette smoke on blood pressure in children and adolescents: a meta-analysis of epidemiologic studies. BMC Pediatr.

[147] Huxley RR, Woodward M (2011). Cigarette smoking as a risk factor for coronary heart disease in women compared with men: a systematic review and meta-analysis of prospective cohort studies. Lancet..

[148] Jayes L, Haslam PL, Gratziou CG, Powell P, Britton J, Vardavas C (2016). SmokeHaz: systematic reviews and meta-analyses of the effects of smoking on respiratory health. Chest..

[149] Koyanagi YN, Matsuo K, Ito H, Wakai K, Nagata C, Nakayama T (2016). Cigarette smoking and the risk of head and neck cancer in the Japanese population: a systematic review and meta-analysis. Jpn J Clin Oncol.

[150] Peters SA, Huxley RR, Woodward M (2013). Smoking as a risk factor for stroke in women compared with men: A systematic review and meta-analysis of 81 cohorts, including 3 980 359 individuals and 42 401 strokes. Stroke..

[151] Sollie M, Bille C (2017). Smoking and mortality in women diagnosed with breast cancer—a systematic review with meta-analysis based on 400,944 breast cancer cases. Gland Surgery.

[152] Sugawara Y, Tsuji I, Mizoue T, Inoue M, Sawada N, Matsuo K (2019). Cigarette smoking and cervical cancer risk: an evaluation based on a systematic review and meta-analysis among Japanese women. Jpn J Clin Oncol.

[153] Schultze A, Kurz H, Stümpflen I, Hafner E (2016). Smoking prevalence among pregnant women from 2007 to 2012 at a tertiary-care hospital. Eur J Pediatr.

[154] Bhanji S, Andrades M, Taj F, Khuwaja AK (2011). Factors related to knowledge and perception of women about smoking: a cross sectional study from a developing country. BMC Womens Health.

[155] Do EK, Green TL, Prom-Wormley EC, Fuemmeler BF (2018). Social determinants of smoke exposure during pregnancy: Findings from waves 1 & 2 of the Population Assessment of Tobacco and Health (PATH) Study. Prev Med Rep.

[156] Bailey BA (2006). Factors predicting pregnancy smoking in Southern Appalachia. Am J Health Behav.

[157] Homish GG, Eiden RD, Leonard KE, Kozlowski LT (2012). Social-environmental factors related to prenatal smoking. Addict Behav.

[158] Schoenaker DAJM, Ploubidis GB, Goodman A, Mishra GD (2017). Factors across the life course predict women’s change in smoking behaviour during pregnancy and in midlife: results from the National Child Development Study. J Epidemiol Community Health.

[159] Abraham M, Alramadhan S, Iniguez C, Duijts L, Jaddoe VW, Den Dekker HT (2017). A systematic review of maternal smoking during pregnancy and fetal measurements with meta-analysis. PLoS One.

[160] Marufu TC, Ahankari A, Coleman T, Lewis S (2015). Maternal smoking and the risk of still birth: systematic review and meta-analysis. BMC Public Health.

[161] McDonnell BP, Regan C (2019). Smoking in pregnancy: pathophysiology of harm and current evidence for monitoring and cessation. Obstet Gynaecol.

[162] McEvoy CT, Spindel ER (2017). Pulmonary Effects of Maternal Smoking on the Fetus and Child: Effects on Lung Development, Respiratory Morbidities, and Life Long Lung Health. Paediatr Respir Rev.

[163] Mathers CD, Loncar D (2006). Projections of global mortality and burden of disease from 2002 to 2030. PLoS Med.

[164] He Y, Shang C, Chaloupka FJ (2018). The association between cigarette affordability and consumption: An update. PLoS One.

[165] Organization WH (2019). European tobacco use: Trends report 2019.

[166] Mann C (2003). Observational research methods. Research design II: cohort, cross sectional, and case-control studies. Emerg Med J.

[167] Setia MS (2016). Methodology Series Module 3: Cross-sectional Studies. Ind J Dermatol.

[168] Prince SA, Cardilli L, Reed JL, Saunders TJ, Kite C, Douillette K (2020). A comparison of self-reported and device measured sedentary behaviour in adults: a systematic review and meta-analysis. Int J Behav Nutr Phys Act.

