# Supplementary material for: National, regional, and global prevalence of cigarette smoking among women/females in the general population: a systematic review and meta-analysis
Source: Environ Health Prev Med. 2021 Jan 8;26:5. doi: 10.1186/s12199-020-00924-y (PMC7796590; doi:10.1186/s12199-020-00924-y)
Supplement: Supplementary file 1 — Additional file 1: Table S1. Population characteristics of the studies reported the prevalence of current and ever cigarette smoking among women. [file 12199_2020_924_MOESM1_ESM.docx]

**Table S1.** Population characteristics of the studies reported the prevalence of current and ever cigarette smoking among women

| **First Author** | **Year data gathering** | **Study Area** | **Population of study** | **Sample size** | **Age** | **Study design** | **Sample method** | **Tools** | **Current Use (%)** | **Ever Use(%)** |
| --- | --- | --- | --- | --- | --- | --- | --- | --- | --- | --- |
| Al-Sahab. et al, 2010[23] | 2006 | Canada | Pregnant women | 76508 | ≥15 | Cross-sectional study | Stratified random | MES | 10.48 | 49 |
| McClave. et al, 2010[24] | 2002-4 | USA | Women of Reproductive Age | 76544 | 18-44 | Case-control study | Purposefully | BRFSS | 24.33 | - |
| Teater. et al, 2010[25] | 2005 | USA | Women in Substance Abuse Treatment | 645 | 14-67 | Cross-sectional study | Voluntarily participated | Self- report | 79 | - |
| Tappin. et al, 2010[26] | 2005 | Scotland | Pregnant women | 52370 | 15-35 | Cross-sectional study | - | Self-reported smoking | 25 | - |
| Mandil. et al, 2010[27] | 2008-9 | Saudi Arabia | University students | 207 | 17-25 | Cross-sectional | Stratified | GYTS | 34.8 | - |
| Al Ghobain. et al, 2011[28] | 2010 | Saudi Arabia | Secondary school students | 1272 | 16-18 | Cross-sectional study | Cluster | GYTS | 8.91 | 31.41 |
| Fawibe. et al, 2011[29] | 2009 | Nigeria | College students | 599 | 16-43 | Cross-sectional study | Randomly | Self- report | 2 | 6.2 |
| Reynolds. et al, 2011[30] | 2001 | USA | Type 1 or Type 2 Diabetes | 1801 | ≥10 | Cross-sectional study | Available sampling | Self-report | 11 | - |
| Blix. et al, 2011[31] | 2004-9 | Norway | Public population | 189940 | 44-61 | Cohort study | Census | Self- report | - | 63 |
| De Santis. et al, 2011[32] | 2008-10 | Italy | Pregnant women | 503 | ≥15 | Cross-sectional study | Available sampling | Self-report | 22.7 | - |
| Muckle. et al, 2011[33] | 2000 | Canada | Pregnant women | 248 | 14-40 | Cross-sectional study | Census | SQIHS | - | 92 |
| Tettamanti. et al, 2011[34] | 2005 | Sweden | Bladder pain symptoms Female | 9336 | 19-47 | Cross-sectional study | Census | Self-report | 20 | 40.65 |
| Lau. et al, 2012[35] | 2009 | Hong Kong | School student | 3169 | 10-17 | Cross-sectional study | Cluster | Self-report | 3.7 | 8.8 |
| Salameh. et al, 2012[36] | 2009-2010 | Lebanon | Public population | 1134 | ≥40 | Cross-sectional study | Cluster | Self- report | 36.8 | 42.9 |
| Peltzer. et al, 2012[37] | 2008 | South Africa | School Adolescents | 4048 | 44-53 | Cross-sectional study | Cluster | GYTS | 10.5 | - |
| Gallopel-Morvan. et al, 2012[38] | - | France | Adult Population | 435 | ≥18 | Cross-sectional study | Randomly | Self-report | - | 32.7 |
| Moeini. et al, 2012[39] | 2010 | Iran | School Students | 573 | 14-20 | Cross-sectional | Randomly | Self-Report | 6.81 | - |
| Jung-Choi. et al, 2012[40] | 2008 | South Korea | Public population | 3068 | 20-92 | Cross-sectional study | Stratified | Self- report | - | 14 |
| Fielder. et al, 2013[41] | 2009-2010 | USA | First year of college | 424 | 18 | Cross-sectional study | Randomly | Self-report | 9 | 18 |
| Jones. et al, 2013[42] | 2010 | USA | Pregnant | 131 | 13-79 | Randomized clinical trial | Randomly | TDS | - | 97 |
| Hattori. et al, 2013[43] | 2000-8 | Japan | Sarcoidosis patients | 237 | ≥20 | Cross-sectional study | Purposefully | ECRHS | 27.9 | 38 |
| Jawad. et al, 2013[44] | 2011-12 | UK | School students | 1212 | 44-56 | Cross-sectional study | Census | GYTS | 3.6 | 16.7 |
| Tamai. et al, 2013[45] | 2003-6 | Japan | Participants referral to hospital | 939 | 20-74 | Cross-sectional study | Voluntarily participated | Self- report | 6.5 | 10.22 |
| Di Giuseppe. et al, 2013[46] | 2003-10 | Sweden | All women | 34101 | 54-89 | Cohort study | Census | Self- report | 23 | 46 |
| Ferrite. et al, 2013[47] | 2006 | Brazil | women as exposed to noise | 1723 | 20-49 | Cross-sectional study | Randomly | Self- report | - | 18.6 |
| Parajuli. et al, 2013[48] | 2007 | Norway | Public population | 302866 | 19-67 | Cohort study | Randomly | Self- report | - | 59.22 |
| Saito. et al, 2013[49] | 2008 | Japan | Public population | 63894 | 40-79 | Cohort study | - | Self- report | 4.82 | 5.52 |
| Lee. et al, 2013[50] | 2007-9 | South Korea | Women of Reproductive Age | 5031 | 20-49 | Cross-sectional study | Stratified multistage clustered probability | Self- report | - | 7.3 |
| Azab. et al, 2013[51] | 2011 | Jordan | Pregnant | 500 | 20- 40 | Cross-sectional study | Randomly | Self- report | 8 | 38.3 |
| Baheiraei. et al, 2014[52] | 2010 | Iran | Women of reproductive | 1359 | 15-49 | Cross-sectional study | Random multistage cluster | Self- report | - | 3.1 |
| Land. et al, 2014[53] | - | USA | Women at High Risk of Breast Cancer nursing students | 13388 | >65 | Cohort study | Randomly | Self- report | 12.5 | 32.5 |
| Czoli. et al, 2014[54] | 2012 | Canada | Youth/young adults | 672 | 16-30 | Cross-sectional | Non-probability | Self-Report | 40.03 | 46.88 |
| Lehmann. et al, 2014[55] | 2008-2013 | Germany | Nursing students | 525 | 16-30 | Case-control study | - | Self- report | - | 41.71 |
| Berg. et al, 2014[56] | 2010 | Georgia | Adolescents/youth | 962 | 15-24 | Cross-sectional | Stratified | GYTS | 11.95 | 32.33 |
| Hossain. et al, 2014[57] | 2011 | Bangladesh | Married Women | 8074 | 18-96 | Cross-sectional | Randomly | Self-Report | 25.1 | 44.1 |
| Jawad MBBS. et al, 2015[58] | 2012 | Jordan | Women of reproductive | 11113 | 15-49 | Cross-sectional study | Cluster | DHS | - | 10.9 |
| Madhu. et al, 2015[59] | 1991-2009 | UK | Women who underwent urodynamic assessment for various LUTS | 11678 | ≥18 | Retrospective study | Purposefully | Self- report | - | 21.2 |
| Al-Zalabani. et al, 2015[60] | 2013 | Saudi Arabia | School students | 1559 | 44-54 | Cross-sectional study | Stratified cluster | GYTS | - | 8.3 |
| Fotiou. et al, 2015[61] | 2014 | Greece | School students | 682 | 15 | Cross-sectional study | Clustered probability | Self- report | 17.3 | 37.1 |
| Ngahane. et al, 2015[62] | 2012-13 | Cameroon | College students | 1579 | 44-94 | Cross-sectional study | Stratified | Self- report | 5.3 | - |
| Liozidou. et al, 2015[63] | 2013-14 | Greece | School Adolescents | 992 | 44-83 | Cross-sectional study | Randomly | Self- report | 16.5 | - |
| Syamlal. et al, 2015[64] | 2008-12 | USA | Worked in the healthcare and social assistance sector | 14912100 | 18-85 | Cross-sectional study | Randomly | Self- report | 24.42 | - |
| Biedermann. et al, 2015[65] | 2012 | Swiss | Patients | 900 | 14-65 | Cohort study | - | Self- report | - | 56 |
| Lange. et al, 2015[66] | 2003-12 | Canada | Pregnant women | 22962 | 18-45 | Cross-sectional study | Randomly | Self- report | 17.7 | - |
| Li. et al, 2015[67] | 2011 | USA | Women of reproductive | 9789 | 18-25 | Cross-sectional study | Randomly | Self- report | 58.43 | 30.3 |
| Taheri. et al, 2015[68] | 2008 | Iran | University students | 519 | ≥18 | Cross-sectional | Census | GHPS | 2.5 | - |
| Veeranki. et al, 2015[69] | 2008 | Madagascar | School Students | 649 | 13-15 | Cross-sectional | Cluster | GYTS | 10.2 | - |
| Iqbal. et al, 2015[70] | 2012 | Pakistan | Public population | 16987 | 15-80 | Cross-sectional | Cluster | GATS | 12.32 | - |
| Kristjansson. et al, 2015[71] | 2015 | Iceland | School students | 1766 | 15-16 | Cross-sectional | - | Self-report | 5.2 | - |
| Chivers. et al, 2016[72] | 2014 | USA | Women of reproductive | 800 | 24-44 | Cross-sectional study | - | MCQ | 50 | - |
| Mazurek. et al, 2016[73] | 2009-13 | UK | Women of reproductive | 30855 | 18-49 | Cross-sectional study | Randomly | Self- report | 12.9 | 17.3 |
| Thrasher. et al, 2016[74] | 2015 | Mexico | Middle school students | 5073 | ≥11 | Cross-sectional study | Stratified random | Self- report | - | 18.19 |
| Westling. et al, 2016[75] | 2014-16 | USA | School students | 559 | 18< | Cross-sectional study | Voluntarily participated | self- report | 8.91 | 21.11 |
| Rennie. et al, 2016[76] | 2014-15 | France | Adolescents | 705 | 11-18 | Cross-sectional | - | self-Report | - | 58.3 |
| Bressler. et al, 2016[77] | 2010-12 | USA | Reproductive-age African-American women | 1654 | 23-35 | Cross-sectional study | Voluntarily participated | CAWI | 19 | 26.66 |
| Jeon. et al, 2016[78] | 2015 | South Korea | Adolescents/adults | 2249 | 13-29 | Cross-sectional | Voluntarily participated | GYTS | 3.92 | 5.16 |
| Xu. et al, 2016[79] | 2016 | China | School Students | 268 | 15-18 | Cross-sectional | Randomly | Self-Report | - | 4.34 |
| Joung. et al, 2016[80] | 2014 | South Korea | School students | 35590 | 11-18 | Cross-sectional | Cluster | Self-report | 4.1 | - |
| Persoskie. et al, 2016[81] | 2014 | USA | School Students | 10645 | 9-19 | Cross-sectional | Cluster | Self-report | - | 21.1 |
| Kruger. et al, 2016[82] | 2013 | South Africa | University students | 2509 | ≥18 | Cross-sectional study | Census | Self- report | 14.8 | 59.1 |
| Nicolaou. et al, 2016[83] | 2009-10 | Greece | Adults | 1628 | 25-64 | Cross-sectional | Randomly | CTUMS | 18.43 | 26.23 |
| Liu. et al, 2017[84] | 2010 | China | Public population | 53082 | 18-70 | Cross-sectional study | Randomly | self- report | 2.6 | 3.4 |
| Ma. et al, 2017[85] | 2012-14 | China | Women refer to hospital | 3581 | ≥15 | Cross-sectional study | Census | Self- report | 3.2 | - |
| Wagner. et al,2017[86] | 2015 | USA | Pregnant women | 445 | ≥15 | Cross-sectional study | Voluntarily participated | Self- report | - | 5.6 |
| McCabe. et al, 2017[87] | 2014 | USA | Adolescents | 3900 | 11-20 | Cross-sectional | Randomly | Self-report | 5.05 | - |
| Milicic. et al, 2017[88] | 2014-15 | Canada | School students | 19909 | 12-18 | Cohort | Purposefully sampled | Self-report | 4.67 | 5.31 |
| Kelemen. et al, 2017[89] | 2010 | USA | African American Cancer women patients | 1365 | >20 | Case-control study | Randomly | Self- report | 15.6 | 43.08 |
| Nemati. et al, 2017[90] | 2006-9 | Iran | Adult Population | 59733 | 15-64 | Cross-sectional study | Stratified cluster random | Self- report | - | 0.8 |
| Karadoğan. et al, 2017[91] | 2015 | Turkey | School teachers | 164 | 21-63 | Cross-sectional study | Randomly | Self- report | 12.8 | 21.95 |
| Balachova. et al, 2010[92] | - | Russia | Childbearing age | 657 | 18-44 | Cross-sectional study | Stratified | Self- report | 49 | - |
| Deacon. et al, 2017[93] | 2010-14 | Australia | Lesbian, bisexual and queer women | 2044 | 16-81 | Cross-sectional study | Convenience sampling | SWASH | 34 | 59 |
| Owolabi. et al, 2017[94] | 2016 | South Africa | Adults | 671 | 18-75 | Cross-sectional | Convenience sampling | WHO STEP wise | 6.5 | - |
| Auf. et al, 2018[95] | 2014-15 | USA | Adolescents | 4225 | 12-19 | Cross-sectional | Cluster probability | Self-report | 70.73 | - |
| Jaber. et al, 2018[96] | 2013-14 | USA | Public population | 3078 | ≥18 | Cross-sectional study | Stratified | SMQRTU | 47.9 | 37.65 |
| Cozier. et al, 2018[97] | 2015 | USA | Black Women | 56552 | 21-69 | Cohort study | Randomly | Self- report | 16 | 18 |
| Melka. et al, 2018[98] | 2015 | Australia | Young Women | 8915 | 19-26 | Cohort study | - | Self- report | 16.75 | 24.73 |
| Zidi. et al, 2018[99] | 2009-16 | Tunisia | Women with Cervical Cancer | 600 | 30-70 | Retrospective study | Randomly | Self- report | - | 30 |
| Duncan. et al, 2018[100] | 2007-8 | Canada | Women of childbearing | 249 | 18–39 | Cross-sectional study | - | Self- report | 81.5 | - |
| Miyazaki. et al, 2018[101] | 2015 | Japan | Adults | 3706 | 18-69 | Cross-sectional | Randomly | Self-report | 8.7 | - |
| Treur. et al, 2018[102] | 2014-15 | Netherlands | Adolescent | 1692 | 11-17 | Cohort | Randomly | Self-report | 10.5 | 19.9 |
| Tolstrup. et al, 2018[103] | 2014 | Denmark | Adolescents | 42773 | 16-19 | Cross-sectional | Census | Self-report | 11 | - |
| Delk. et al, 2018[104] | 2015-16 | USA | Adolescents | 1434 | 11-18 | Cross-sectional | Voluntary participation | Self-report | 1.81 | 12.48 |
| Karadoğan. et al, 2018[105] | 2017 | Turkey | University students | 1029 | ≥18 | Cross-sectional | Randomly | Self-Report | 46 | - |
| Abdollahpour. et al, 2019[106] | 2013-15 | Iran | Women of reproductive | 544 | 15-50 | Cross-sectional study | Randomly | Self- report | 4.04 | 5.51 |
| Wang. et al, 2019[107] | 2016-17 | China | Adults | 21786 | ≥18 | Cross-sectional | Randomly | Self-Report | - | 0.3 |
| Pinkas. et al, 2019[108] | 2019 | Poland | Public population | 527 | ≥15 | Cross-sectional study | Randomly | GATS | - | 20.51 |
| Kondracki. et al, 2019[109] | 2016 | USA | Pregnant women | 275101 | ≥14 | Cross-sectional study | Randomly | Self- report | 7.1 | 9.4 |
| Zhu. et al, 2019[110] | 2017-18 | China | School personnel | 63474 | ≥25 | Cross-sectional study | Stratified cluster | GSPS | 0.5 | - |
| Desai. et al, 2019[111] | 2010-11 | South Africa | Students of school dropouts | 1716 | 13-20 | Cross-sectional study | Non-probability | Self- report | 33.9 | - |
| Gallopel-Morvan. et al, 2019[112] | 2013 | France | Adult Population | 490 | 15-30 | Cross-sectional study | Randomly | Self- report | - | 48 |
| Jawad. et al, 2019[113] | 2011-12 | UK | Pregnant women | 1044 | ≥14 | Cross-sectional study | Convenience sampling | Self- report | 5 | 61.69 |
| Praeger. et al, 2019[114] | 2013 | Australia | Lesbian and | 11911 | 14-84 | Cross-sectional study | Randomly | Self- report | 12.85 | 35.25 |
| Barrientos-Gutierrez. et al, 2019[115] | 2015-16 | Mexico | Adolescents | 4143 | 11-18 | Cross-sectional | Randomly | Self-report | 11 | 45 |
| Park. et al, 2019[116] | 2014-16 | South Korea | Adults | 8493 | ≥19 | Cross-sectional | Stratified | Self - report | 2.6 |  |
| Kilibarda. et al, 2019[117] | 2017 | Serbia | School Students | 1637 | 13-15 | Cross-sectional | Cluster | Self - report | 11.3 | - |
| Chang. et al, 2019[118] | 2016 | South Korea | Adults | 18859 | ≥19 | Cross-sectional | Stratified | Self - report | 2.73 | - |
| Soule. et al, 2019[119] | 2017 | USA | College students | 32136 | ≥18 | Cross-sectional | Stratified | NCHA-II | 7.7 | - |
| Zhao. et al, 2019[120] | 2013-14 | China | Adults | 16143 | ≥15 | Cross-sectional | Clustered | Self-report | 2.1 | - |
| Jiang. et al, 2019[121] | 2016 | Hong Kong | Young adults | 649 | 18-35 | Cross-sectional | Voluntary participation | Self-report | 48.8 | - |
| Rollins. et al, 2020[122] | 2015-18 | USA | Pregnant women | 1365 | 16-45 | Cross-sectional study | Voluntarily participated | Self- report | 27.3 | - |
| Cho. et al, 2020[123] | 2011-16 | India | Public population | 1298933 | ≥18 | Cross-sectional study | - | Self- report | - | 14.9 |
| Vatankhah. et al, 2020[124] | 2017 | Iran | Public population | 2888 | ≥65 | Cross-sectional study | Cluster | Self- report | 5.75 | - |
| Walker. et al, 2020[125] | 2014-19 | New Zealand | School students | 68643 | 14-15 | Cross-sectional study | Voluntarily participated | Self- report | - | 24.66 |
| Parekh. et al, 2020[126] | 2019 | USA | Young Adults | 85772 | 18-44 | Cross-sectional | Publicly available data | BRFSS | 6.68 | 8.15 |
| Shan. et al, 2020[127] | 2016-17 | Canada | School students | 16844 | 12-18 | Cross-sectional | Randomly | Self- report | 4.38 | - |
| Chun. et al, 2020[128] | 2017 | South Korea | Adolescents | 29830 | 13-18 | Cross-sectional | Randomly | Self- report | 3.05 | 6.83 |
| Roys. et al, 2020[129] | 2014-16 | USA | College students | 885 | ≥18 | Cross-sectional | - | Self- report | 4.07 | - |
| Roberts. et al, 2020[130] | 2013-15 | USA | Adults | 13359 | ≥18 | Cohort | Stratified | Self- report | 31.19 | - |
| Kava. et al, 2020[131] | 2017 | USA | Adult Employees | 99569 | ≥18 | Cross-sectional | Randomly | Self- report | 12 | - |

**Abbreviations:** **NCHA-II:** The National College Health Assessment-II; **GATS:** The Global Adult Tobacco Survey; **GYTS:** Global youth tobacco survey; **MES:** Maternity Experience Survey; **BRFSS:** Behavior Risk Factor Surveillance System; **TDS**: Tobacco dependence screener; **SQIHS**: Santé Quebec Inuit Health Survey; **ECRHS:** European Community Respiratory Health Survey; **DHS:** Demographic and Health survey; **MCQ:** Monetary Choice Questionnaire; **CAWI:** Self-administered computer-assisted web interviewing; **GHPS:** Global Health Professional Survey; **CTUMS:** Canadian Tobacco Use Monitoring Survey; **SWASH:** Sydney Women and Sexual Health survey; **SMQRTU:**  [Smoking–Recent Tobacco Use;](http://www.nber.org/nhanes/2005_2006/downloads/smqrtu_d.pdf) **GSPS:** Global School Personnel Survey.
