# Supplementary material for: National, regional, and global prevalence of cigarette smoking among women/females in the general population: a systematic review and meta-analysis
Source: Environ Health Prev Med. 2021 Jan 8;26:5. doi: 10.1186/s12199-020-00924-y (PMC7796590; doi:10.1186/s12199-020-00924-y)
Supplement: Supplementary file 2 — Additional file 2:. Search Strategy. [file 12199_2020_924_MOESM2_ESM.docx]

| **Search Strategy** |
| --- |
| ***Scopus***  ( TITLE ( "Cigarette*" ) OR TITLE ( "Smoking" ) ) AND ( LIMIT-TO ( DOCTYPE , "ar" ) ) AND ( LIMIT-TO ( LANGUAGE , "English" ) ) AND ( LIMIT-TO ( PUBYEAR , 2020 ) OR LIMIT-TO ( PUBYEAR , 2019 ) OR LIMIT-TO ( PUBYEAR , 2018 ) OR LIMIT-TO ( PUBYEAR , 2017 ) OR LIMIT-TO ( PUBYEAR , 2016 ) OR LIMIT-TO ( PUBYEAR , 2015 ) OR LIMIT-TO ( PUBYEAR , 2014 ) OR LIMIT-TO ( PUBYEAR , 2013 ) OR LIMIT-TO ( PUBYEAR , 2012 ) OR LIMIT-TO ( PUBYEAR , 2011 ) OR LIMIT-TO ( PUBYEAR , 2010 ) ) |
| ***Web of Science***  (from Web of Science Core Collection)  ("Cigarette* ") *OR* **TITLE:** ("Smoking") **Refined by:** **LANGUAGES:** ( ENGLISH ) AND **DOCUMENT TYPES:** ( ARTICLE ) AND **PUBLICATION YEARS:** ( 2020 OR 2019 OR 2018 OR 2017 OR 2016 OR 2015 OR 2014 OR 2013 OR 2012 OR 2011 OR 2010 ) |
| ***PubMed*** Search: ("Cigarette*"[Title]) OR ("Smoking"[Title]) Filters: Free full text, Full text, Journal Article, Humans, English |
| **Ovid**  ((Cigarette or smoking).ti. |
