# Supplementary figures and images for: National, regional, and global prevalence of cigarette smoking among women/females in the general population: a systematic review and meta-analysis
Source: Environ Health Prev Med. 2021 Jan 8;26:5. doi: 10.1186/s12199-020-00924-y (PMC7796590; doi:10.1186/s12199-020-00924-y)

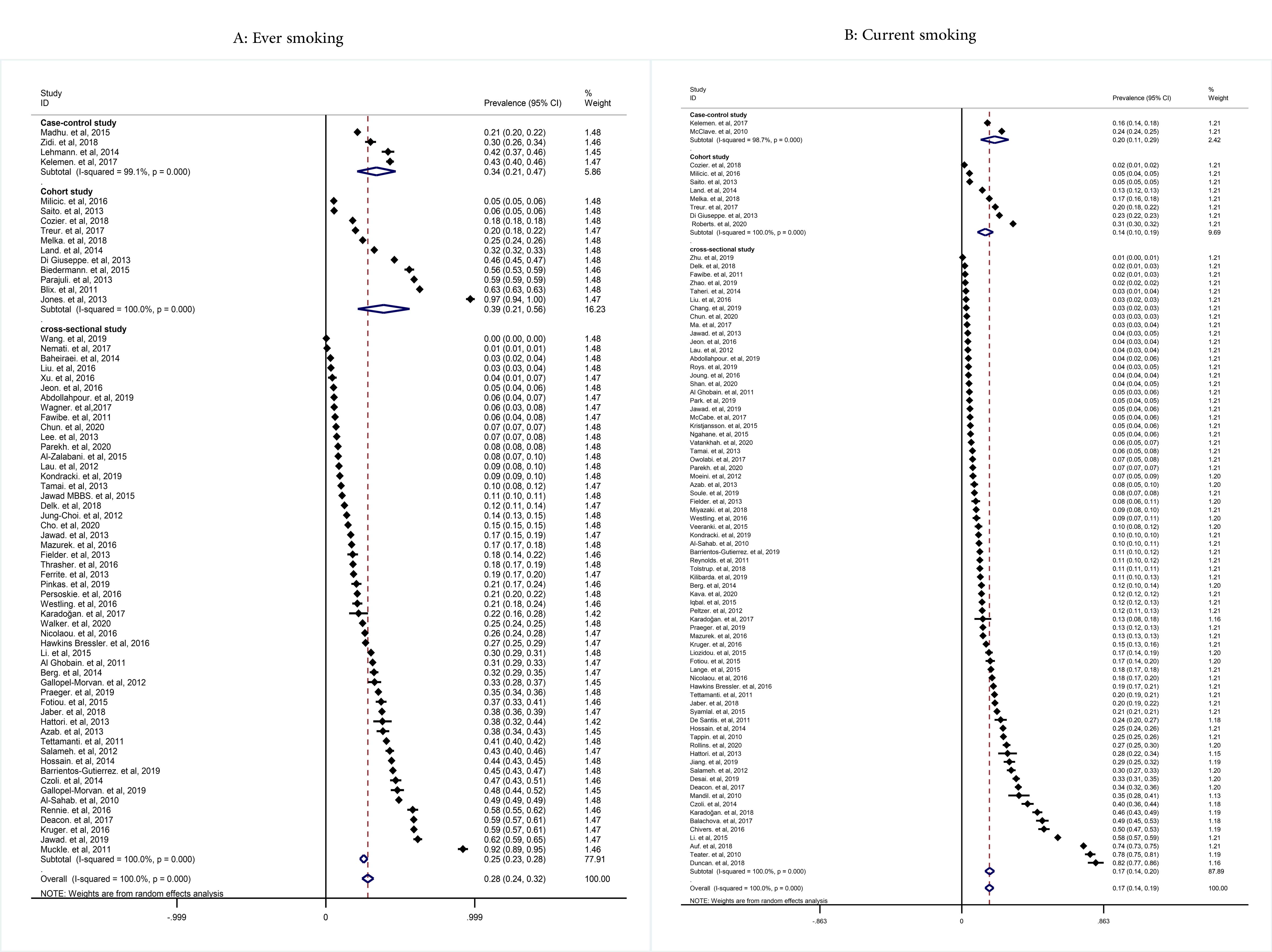

Supplement: Supplementary file 4 — Additional file 4: Fig S1. Pooled ever and current smoking prevalence in women by study design. [file 12199_2020_924_MOESM4_ESM.jpg]

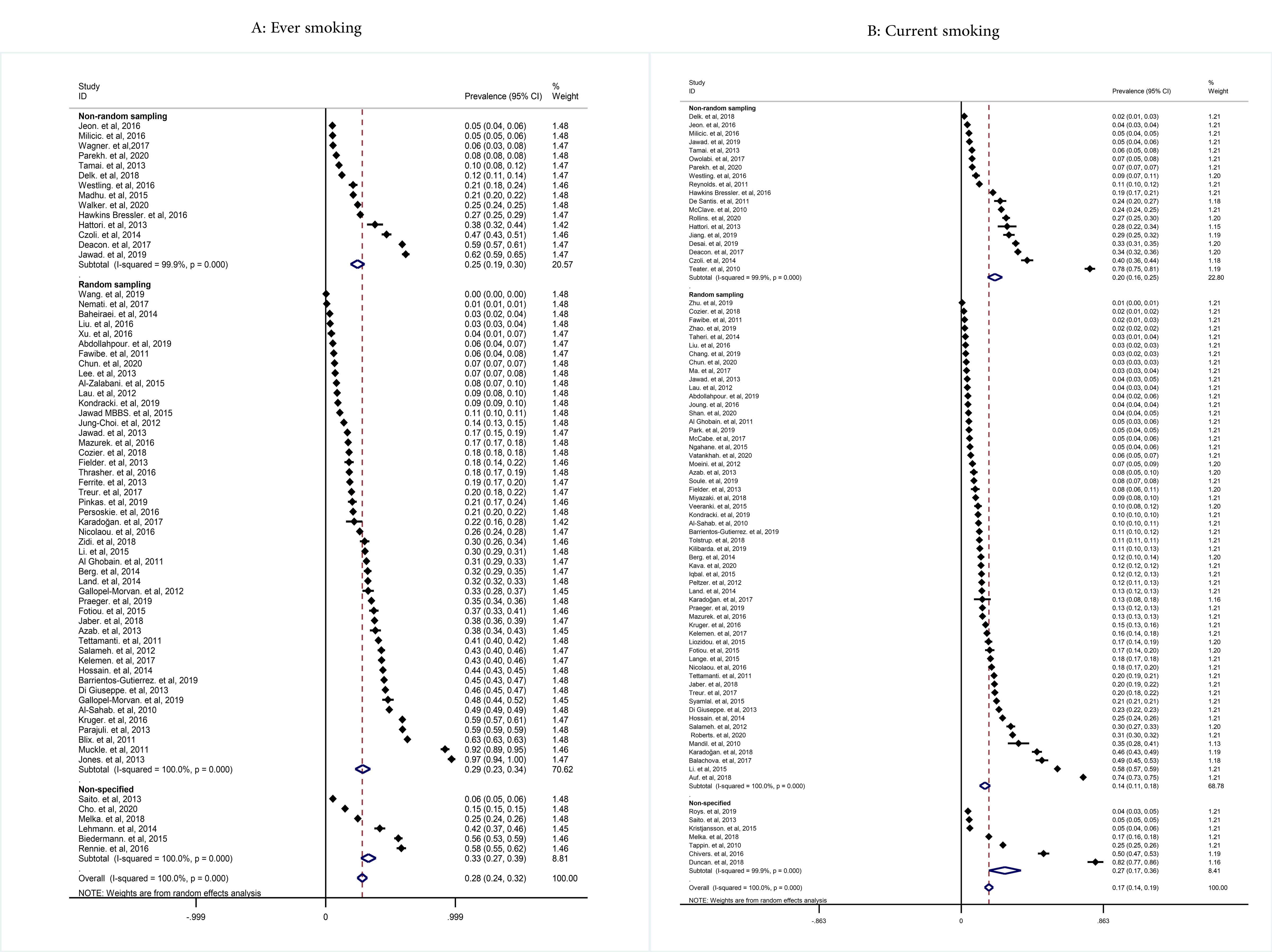

Supplement: Supplementary file 5 — Additional file 5: Fig S2. Pooled ever and current smoking prevalence in women by sampling method. [file 12199_2020_924_MOESM5_ESM.jpg]

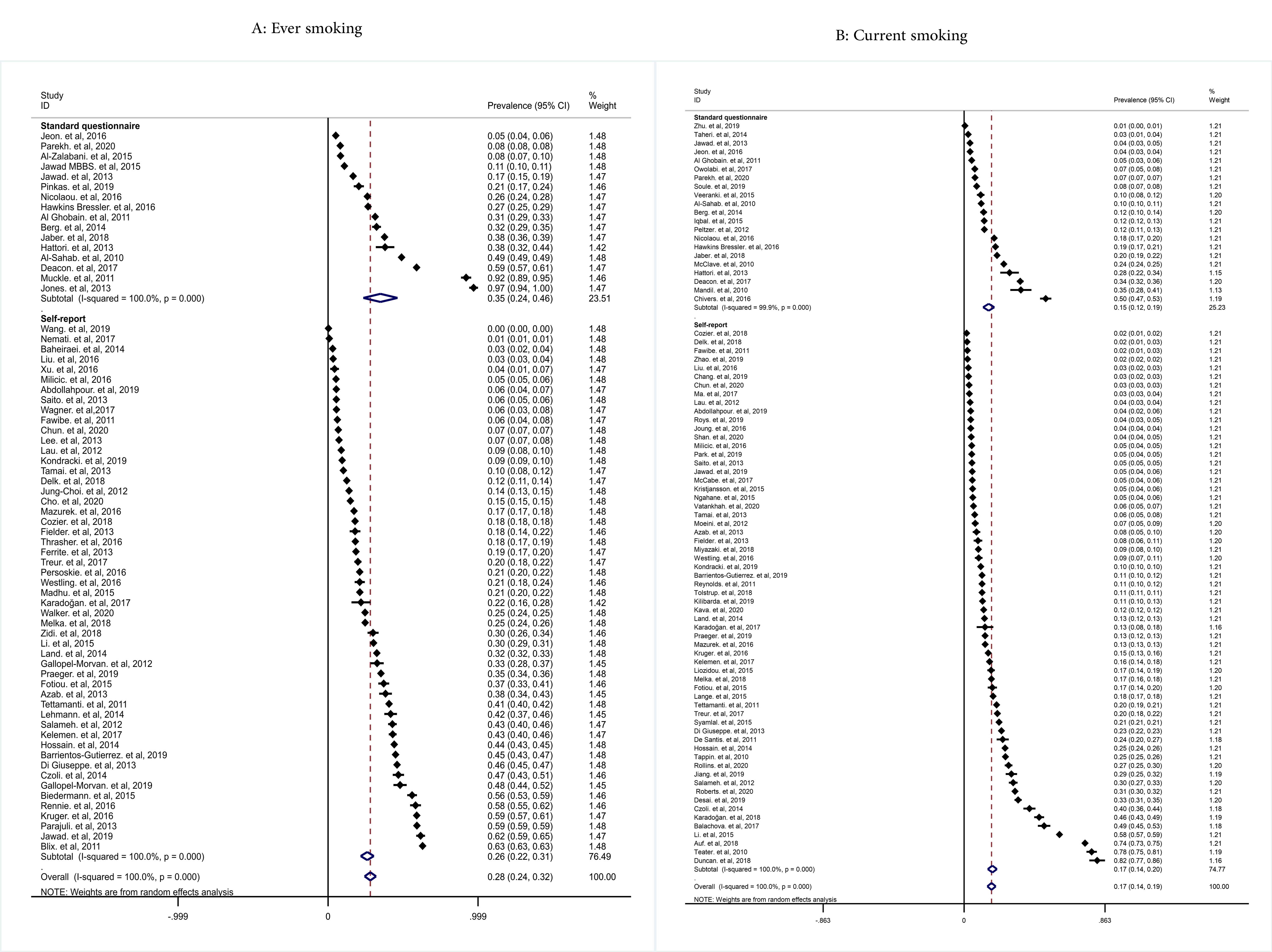

Supplement: Supplementary file 6 — Additional file 6: Fig S3. Pooled ever and current smoking prevalence in women by tools assessment smoking. [file 12199_2020_924_MOESM6_ESM.jpg]

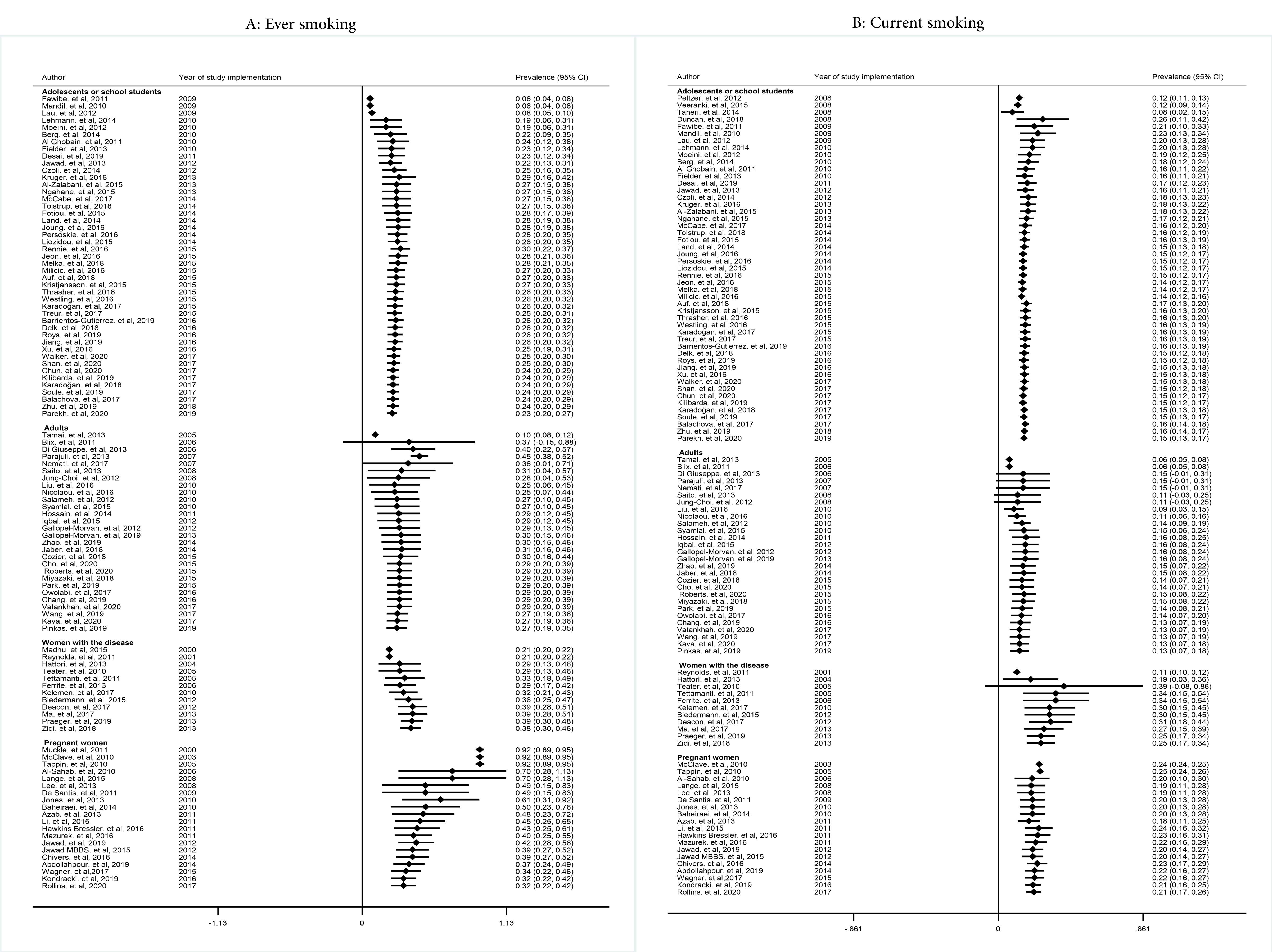

Supplement: Supplementary file 7 — Additional file 7: Fig S4. Cumulative meta-analysis of ever and current smoking prevalence among women by study population. [file 12199_2020_924_MOESM7_ESM.jpg]

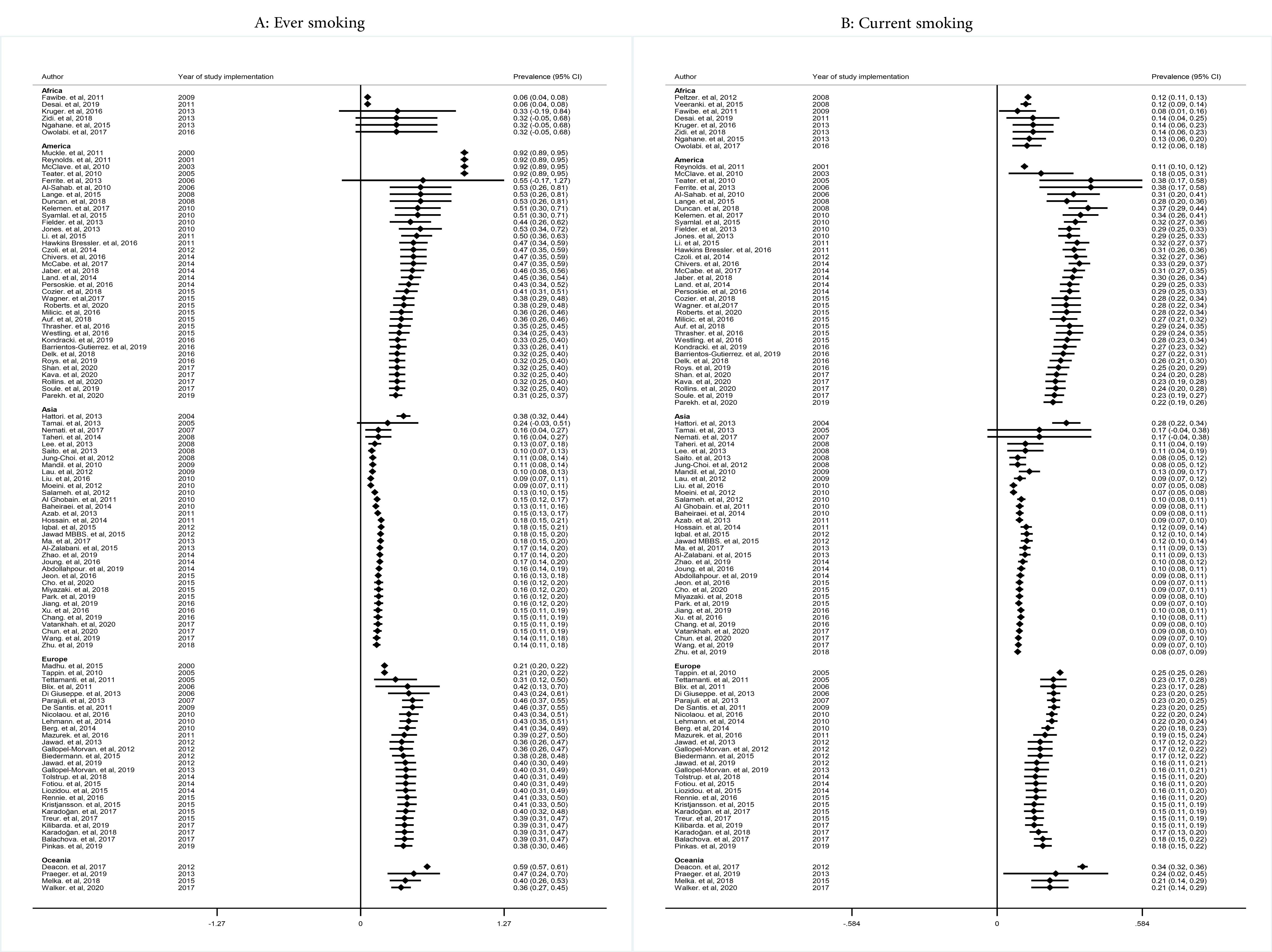

Supplement: Supplementary file 8 — Additional file 8: Fig S5. Cumulative meta-analysis of ever and current smoking prevalence among women by continent. [file 12199_2020_924_MOESM8_ESM.jpg]

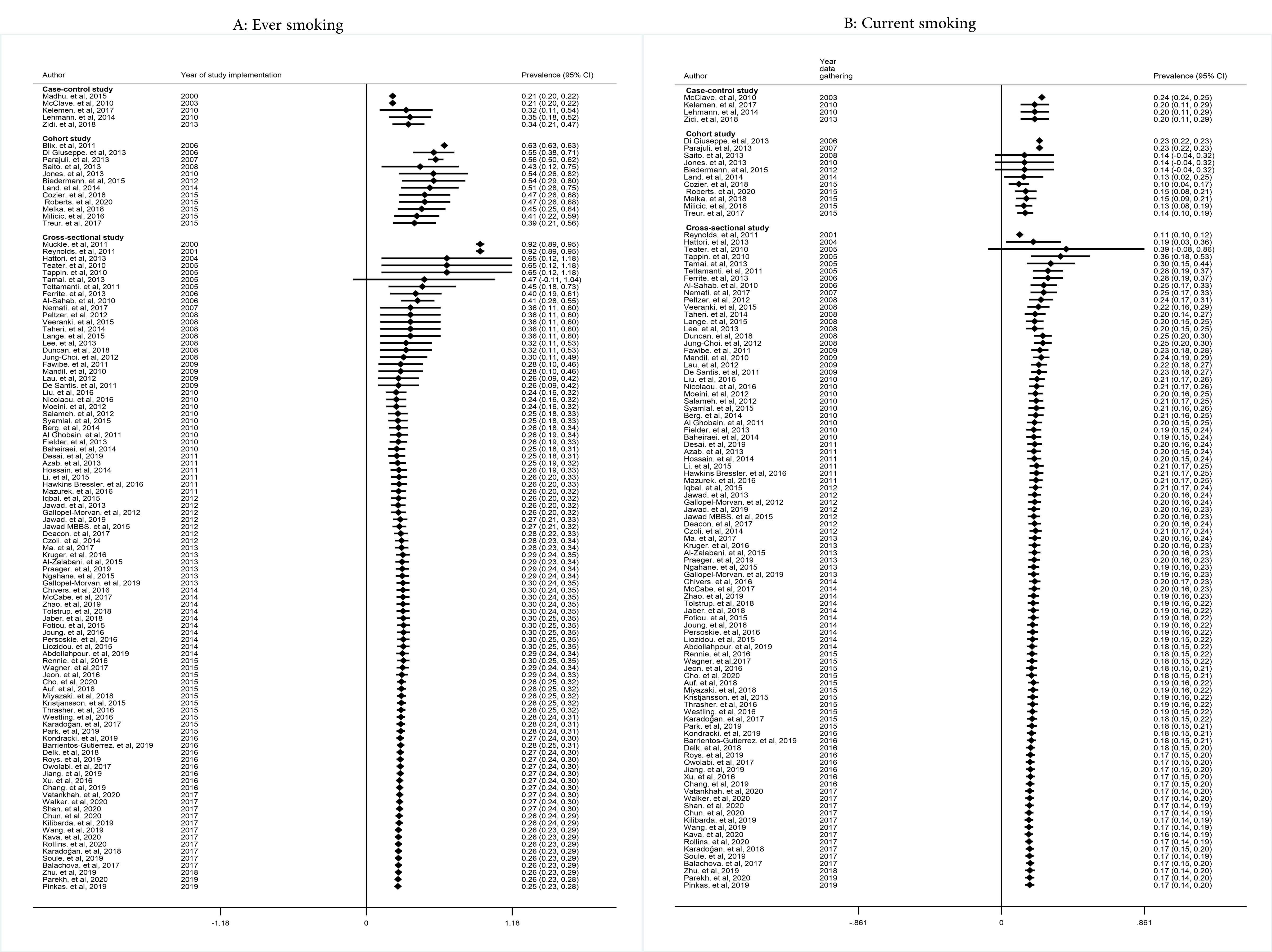

Supplement: Supplementary file 9 — Additional file 9: Fig S6. Cumulative meta-analysis of ever and current smoking prevalence among women by study design. [file 12199_2020_924_MOESM9_ESM.jpg]

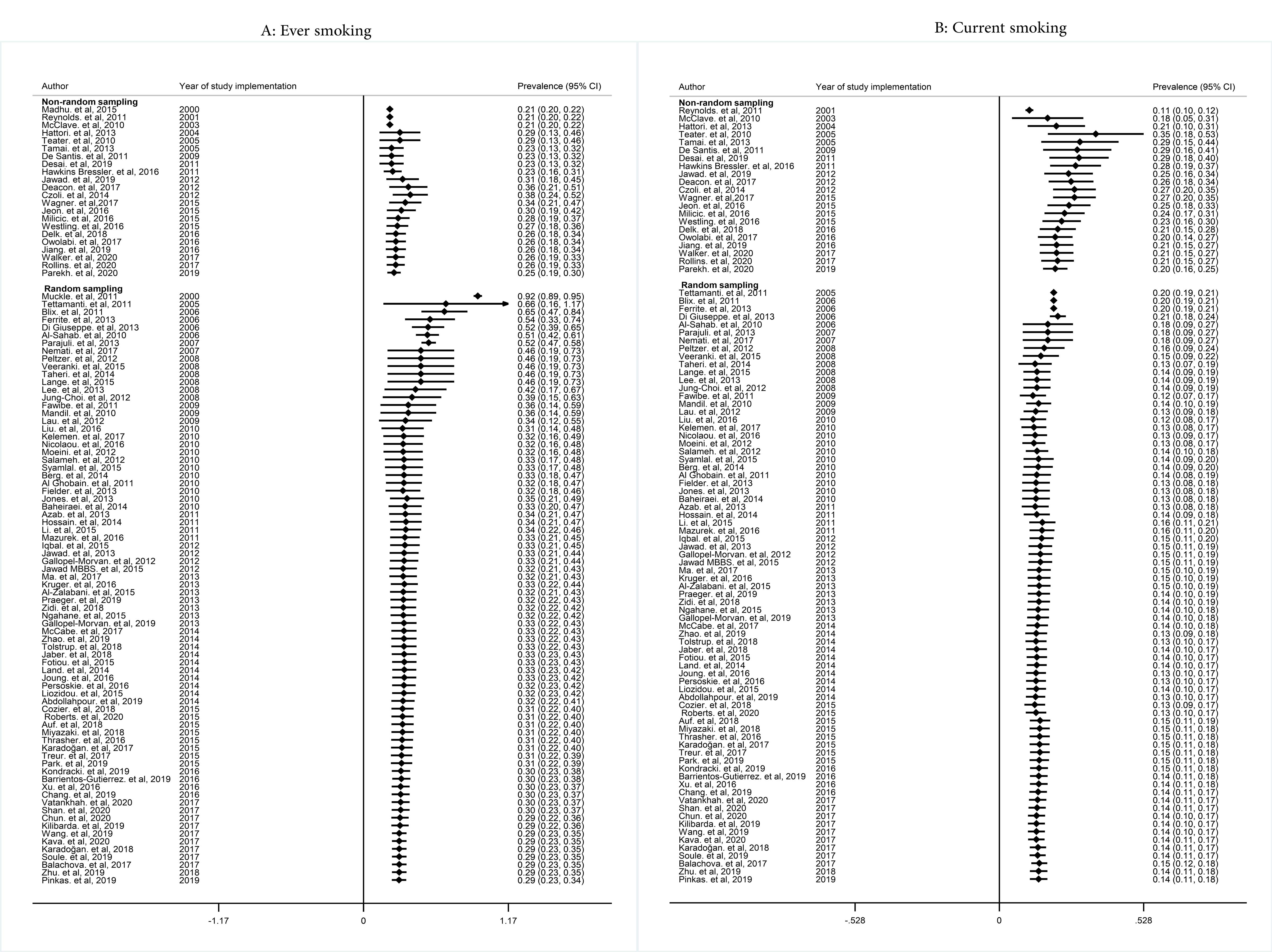

Supplement: Supplementary file 10 — Additional file 10: Fig S7. Cumulative meta-analysis of ever and current smoking prevalence among women by sampling method. [file 12199_2020_924_MOESM10_ESM.jpg]

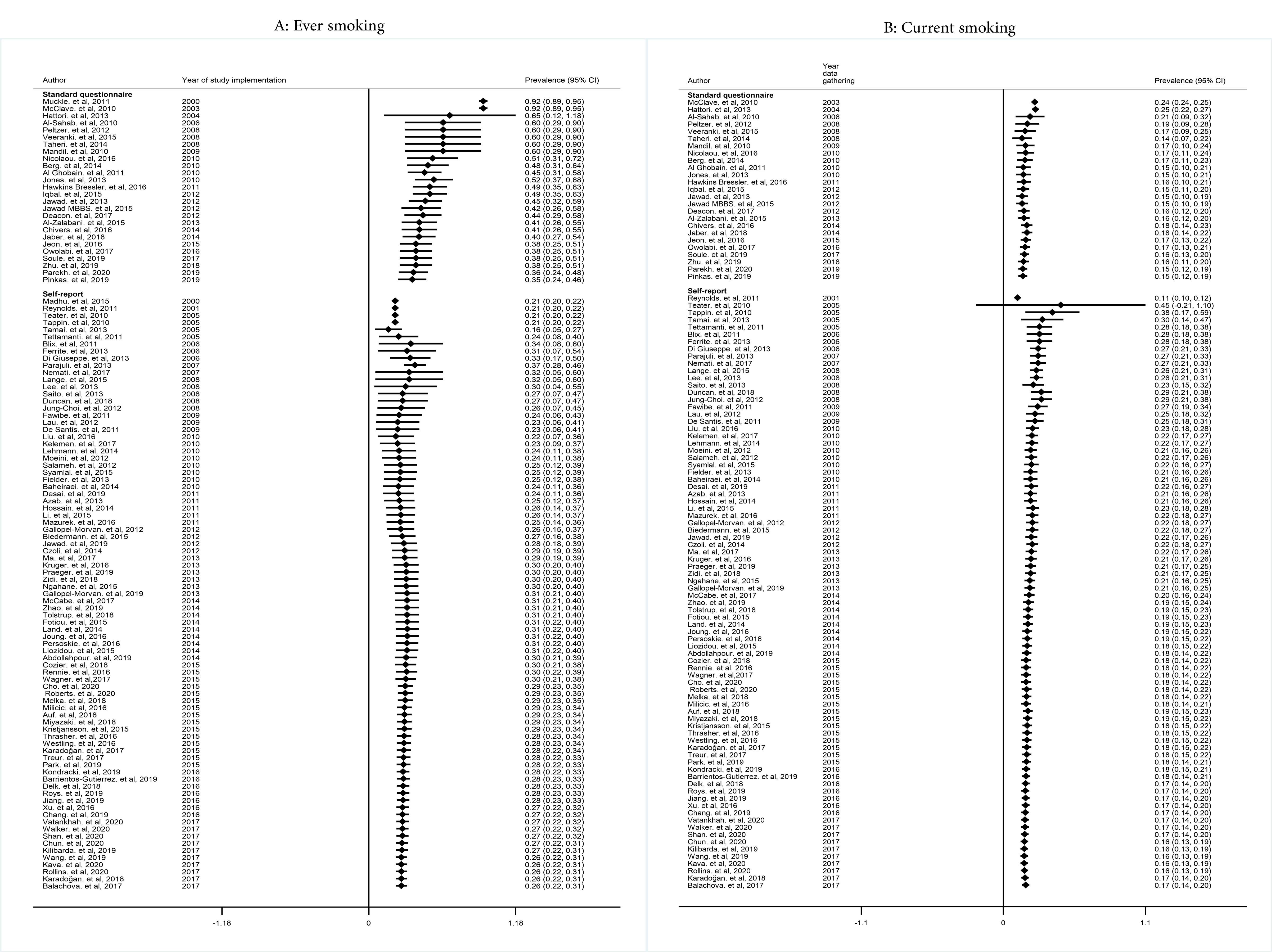

Supplement: Supplementary file 11 — Additional file 11: Fig S8. Cumulative meta-analysis of ever and current smoking prevalence among women tools assessment smoking. [file 12199_2020_924_MOESM11_ESM.jpg]
